# *In silico* analysis of deleterious SNPs of human *MTUS1* gene and their impacts on subsequent protein structure and function

**DOI:** 10.1371/journal.pone.0252932

**Published:** 2021-06-14

**Authors:** Liza Teresa Rozario, Tanima Sharker, Tasnin Akter Nila

**Affiliations:** 1 Department of Biochemistry and Molecular Biology, Noakhali Science and Technology University, Noakhali, Bangladesh; 2 Department of Biochemistry and Molecular Biology, University of Dhaka, Dhaka, Bangladesh; Mayo Clinic Arizona, UNITED STATES

## Abstract

The mitochondrial tumor suppressor 1 (*MTUS1*) gene acts as a crucial tumor suppressor by inhibiting growth and proliferation of eukaryotic cells including tumor cell lines. Down regulation of *MTUS1* gene has been implicated in a wide range of cancers as well as various human diseases. Alteration through nsSNPs can potentially damage the structure and/or function of the protein. As characterization of functional SNPs in such disease linked genes is a major challenge, it is feasible to analyze putative functional SNPs prior to performing larger population studies. Hence, in this *in silico* study we differentiated the potentially harmful nsSNPs of the *MTUS1* gene from the neutral ones by using various sequence and structure based bioinformatic tools. In a total of 215 nsSNPs, 9 were found to be most likely to exert deleterious effect using 7 prediction tools. From which, 5nsSNPs (S1259L, E960K, P503T, L1084V and L1143Q) were selected as potentially damaging due to their presence in the highly conserved region and ability to decrease protein stability. In fact, 2 nsSNPs (S1259L and E960K) among these 5 were found to be individually associated with two distinctive cancers named Stomach adenocarcinoma and Uterine corpus endometrial carcinoma. As this is the first comprehensive study analyzing the functional nsSNPs of *MTUS1*, the results of the current study would certainly be helpful in future prospects concerning large population-based studies as well as drug discovery, especially developing individualized medicine.

## Introduction

Cancer is known as one of the most dreadful and enigmatic diseases in today’s world. Continuous researches are going on to find out new approaches to combat cancers. Among these, identification and characterization of tumor suppressor genes play a crucial role as the loss of tumor suppressor activity is an important benchmark of cancer [1].

Our study focuses on *MTUS1* gene that has been reported as a tumor suppressor gene at a chromosomal position 8p22. This gene encodes a family of microtubule-associated proteins named Microtubule-Associated Scaffold Protein 1(*MTUS1*, previously known as mitochondrial tumor suppressor 1 and microtubule associated tumor suppressor 1) which interacts with angiotensin II type 2 (AT2) receptor. Dispersing over 112 kb on the chromosome, the gene incorporates 17 exons, alternative splicing of which gives onto three major transcripts entitled as ATIP1, ATIP3 and ATIP4. Among them the transcripts of ATIP3 entail three variants- ATIP2, ATIP3a and ATIP3b [2,3]. These three transcripts of ATIP (Angiotensin II receptor-interacting proteins) show diverse tissue distribution along with subcellular compartmentalization, signifying their involvement in various cellular mechanisms [3]. The interaction of ATIP1 with AT2 receptors induces apoptosis and inhibits cell proliferation as well as involves in cell division and migrations [4]. ATIP3 controls critical steps of mitotic cell division reflecting its roles in cell cycle regulation [5].

Furthermore, *MTUS1* gene has been reported to be underexpressed in various types of malignancies including ovarian, breast, head-and-neck, pancreas, colon, bladder and lung cancers [3,5]. The rationality for the lower expression of this gene in various cancers has been implied to mutations. Previous findings have revealed the correlation of mutation and reduced expression of *MTUS1* gene with hepatocellular and squamous cell carcinoma of the tongue accordingly [6,7]. Ovarian carcinoma survival rate also increases after the restoration of ATIP3 which prevents tumor growth via down-regulation of the ERK/EMT pathway [8].

Among different types of mutations single nucleotide polymorphisms (SNPs) account 90% genetic variations in human. SNPs change single base pair in alleles which are the most common form of disparities in DNA sequence [9]. SNPs that alter the encoded amino acids are referred to as nonsynonymous SNPs (nsSNPs), which can influence subsequent structure and/or function of the protein with either neutral or deleterious effects. These nsSNPs are accountable for about half of all genetic variations related to human diseases [10,11].

As human genome contain vast number of genetic polymorphisms, massive investigations are indispensable to explore the significance of each of them along with their association to disease susceptibilities as well as individualized drug designing [10]. However, to lessen this enormous effort numerous computational based methods have been developed to identify prospective and potentially significant variants before testing *in vitro* or *in vivo* conditions. In this situation *in silico* approach is a convenient way to distinguish the deleterious SNPs from neutral ones using particular algorithms. The overall effect of polymorphism including functional and structural alterations can also be analyzed through several databases.

In present study, we use public datasets and freely accessible bioinformatics tools to identify the most deleterious nsSNPs of the *MTUS1* gene and to analyze consequent protein structure and functions. Here, our focus is not only establishing the structural configuration but also finding the association of different cancers with particular nsSNPs. As no effort has been noticed till date, it is the first comprehensive study to analyze the SNPs of the *MTUS1* gene systematically which will be helpful in future extensive studies in this regard.

## Methods

### Retrieval of SNPs

Data related to human *MTUS1* gene and its protein sequence (FASTA format) was collected from NCBI (https://www.ncbi.nlm.nih.gov/) and UniProtKB (http://www.uniprot.org/uniprot/) respectively. SNPs located in *MTUS1* gene were retrieved from dbSNP database (http://www.ncbi.nlm.nih.gov/SNP/) [12].

### Identification and prediction of the effect of deleterious SNPs

To analyze the functional and structural consequences of deleterious SNPs of *MTUS1* gene; SIFT, SNAP2, Align GVGD, PolyPhen-2, PROVEAN, PhD-SNP and PANTHER were used sequentially.

SIFT (Sorting Intolerant from Tolerant) (https://sift.bii.a-star.edu.sg/) determines the deleterious (probability score <0.05) and tolerated SNPs (probability score ≥ 0.05) based on sequence homology. This prediction helps to analyze the effect of amino acid variation on the phenotypic and functional changes upon protein molecule. The rsIDs retrieved from dbSNP were used as a input query for this server [13].

SNAP2 (Screening for Nonacceptable Polymorphisms) (https://www.rostlab.org/services/SNAP/) differentiates between effect and neutral variants by scrutinizing a variety of sequence and variant features. For SNAP2, FASTA format of protein sequences was used as input query. The result was obtained as a score, ranges from −100 (strong neutral prediction) to +100 (strong effect prediction) that reveals the likelihood of specific mutation to alter the native protein function with expected accuracy [14].

Align GVGD (http://agvgd.hci.utah.edu/) web server predicts whether missense substitution is deleterious or neutral. This prediction emphasizes on protein multiple sequence alignments and biophysical characteristics of amino acids. It forms a spectrum of classified variants (C0, C15, C25, C35, C45, C55, C65) where C65 is most likely to interfere with function and C15 is less likely to affect [15]. Here, FASTA sequence of protein and amino acid substitution were used as input file.

For exploring the possible effect of an amino acid substitution on the structure and function of protein, PolyPhen-2 (Polymorphism Phenotyping v2) (http://genetics.bwh.harvard.edu/pph2/) was used. As input query for PolyPhen-2; protein sequence, database ID/ accession number and details of amino acids substitution were given to the server. The score of PolyPhen-2 ranges from 0.0 to 1.0 that indicates the particular amino acid substitution as tolerated or deleterious respectively [16].

By assessing the single amino acid substitutions PROVEAN (Protein variation effect analyzer) (http://provean.jcvi.org/index.php) predicts the functional impact of protein sequence variations as ‘deleterious’ or ‘neutral’. FASTA format sequence with substitutions predicted by the SIFT server was used as an input. If the PROVEAN score is ≤ 2.5 the protein variant is predicted to have a deleterious effect, otherwise the variant is predicted to have a neutral effect [17].

PANTHER cSNP (Protein analysis through evolutionary relationship-codingSNP) (http://pantherdb.org/tools/csnpScoreForm.jsp) classification system is established on evolutionary relationship, molecular functions and their interactions with other proteins. This tool gives position specific evolutionary conservation (PSEC) scores by estimating alignment of evolutionarily related various proteins. Plain protein sequence, amino acid variants and human organism were used as input query for this prediction [18].

PhD-SNP (Predictor of human deleterious single nucleotide polymorphism) (https://snps.biofold.org/phd-snp/phd-snp.html) classifies single point protein mutation as disease-related or as neutral polymorphism based on Support Vector Machine (SVM) method. The required input query was protein sequence, position of mutation and mutated residue [19].

### Prediction of protein stability change by I-Mutant Suite

I-Mutant Suite (http://gpcr2.biocomp.unibo.it/cgi/predictors/I-Mutant3.0/I-Mutant3.0.cgi) predicts the changes in protein stability due to single point mutation. Both protein sequence and protein structure can be used as input query. Upon mutation this tool calculates the sign of the protein stability changes and related Delta Delta G values [20].

### Estimation of conservation profile by ConSurf

Based on the phylogenetic relations between homologous sequences, the ConSurf (https://consurf.tau.ac.il/) server calculates the evolutionary conservation of amino acid positions in a protein. The evolutionary rate of an amino (or nucleic) acid position is strongly dependent on its structural and functional importance and is computed by using either an empirical Bayesian method or a maximum likelihood (ML) method. It uses a color scheme to present conservation score from 1 to 9 which is classified into variable, average and highly conserved. FASTA format of protein sequence was used as input query [21].

### Prediction of solvent accessibility by NetsurfP-2.0

NetSurfP-2.0 (http://www.cbs.dtu.dk/services/NetSurfP/) server is used to predict the surface accessibility, secondary structure, disorder, and phi/psi dihedral angles of amino acids in an amino acid sequence. The FASTA sequence of MTUS1 protein was submitted to NetSurfP and the output of this server revealed buried and exposed region in protein structure [22].

### Analysis of structural effect of nsSNPs

HOPE (Have (y) Our Protein Explained) (https://www3.cmbi.umcn.nl/hope/) assumes the effects of the mutation on the protein structure and the corresponding function. It produces a report, complete with results, figures and animations by collecting and combining available information from a series of web services and databases. Protein sequence and mutation were used as input query for HOPE [23].

### Prediction of protein-protein interactions

STRING (Search Tool for the Retrieval of Interacting Genes/Proteins) (https://string-db.org/cgi/input?sessionId=croExd7fYyEe&input_page_active_form=single_sequence) database gives an important assessment and integration of protein-protein interaction as well as association from databases of physical interaction and databases of curated biological pathway knowledge. Protein sequence was used as input query for this tool [24].

### Prediction and Evaluation of 3D structure of MTUS1 protein

I-TASSER (Iterative Threading ASSEmbly Refinement) (https://zhanglab.dcmb.med.umich.edu/I-TASSER/) is a hierarchical approach for protein structure prediction as well as a structure-based function annotation. It identifies structural templates from the PDB to provide the most appropriate protein structure and usually provides the top 5 models of the targeted protein. The FASTA sequence of MTUS1 was the input file for this server [25].

SWISS-MODEL (https://swissmodel.expasy.org/) is a fully automated protein structure homology-modeling server that uses the updated UniProtKB proteome for target-template alignment. As an input query FASTA sequence was used here. Not only the prediction of structures but also the validation of predicted structures can be estimated through the favored region of Ramachandran plot, QMEAN, and Molprobity score provided by this server [26].

PROCHECK (https://servicesn.mbi.ucla.edu/PROCHECK/) evaluates the stereochemical quality of a protein structure by analyzing residue-by-residue geometry and overall structural geometry [27]. ERRAT (https://servicesn.mbi.ucla.edu/ERRAT/) validates overall model quality by the statistical relationship of non-bonded interactions between different types of atoms based on characteristic atomic interaction. In both cases, predicted models in PDB format was used as an input query [28].

ProSA-web (https://prosa.services.came.sbg.ac.at/prosa.php) is also widely used for the refinement and validation of the experimental protein structure. Predicted structures in PDB format was used for the input query for the estimation of the model quality of the protein [29].

### Three-Dimensional modeling of the mutated protein

Point mutation at the particular position in the native protein sequence was made and SWISS-MODEL (https://swissmodel.expasy.org/) was used for the structural analysis of the mutated protein. The mutated model was then analyzed in TM-align tool (https://zhanglab.dcmb.med.umich.edu/TM-align/) that gives template modeling-score (TM score) and root mean square deviation (RMSD) value for the comparison of protein structures based on the superimposition of the structures to find the structural similarity. In this server, TM-score ranges from 0 to 1, where 1 denotes a perfect match between two structures. In detail, 0.0 < TM-score < 0.30 means random structural similarity, whereas 0.5 < TM-score < 1.00 means both structures are in the same fold [30].

### Identification of cancer association with nsSNPs

cBioPortal (https://www.cbioportal.org/) is an open-access database which allows exploration, visualization and analyzation of multidimensional cancer genomics data. Distribution of *MTUS1* gene mutations in the lollipop plot for mutation frequency in the server was searched to find association of the specific nsSNPs with cancer [31].

canSAR Black (https://cansarblack.icr.ac.uk/) is an integrative translational research and drug discovery web resource for oncology. The mutation profile for *MTUS1* provided by this database was searched for finding specific cancers linked to deleterious nsSNPs [32].

### Analysis of gene expression and overall survival rate

GEPIA (Gene Expression Profiling Interactive Analysis) (http://gepia.cancer-pku.cn/) is an interactive database that is used to analyze the RNA sequencing expression data from the Cancer Genome Atlas (TCGA) and the Genotype-Tissue Expression (GTEx) projects. It provides expression profiles of a given gene in dot plots or box plots and survival analysis by using log-rank test. For both analysis the name of the candidate gene (*MTUS1*) was used as input and specific cancer name (i.e., STAD, UCEC) was selected [33].

### Analysis of correlation of gene expression and mutation

muTarget (https://www.mutarget.com/) is a tool based on the TCGA that correlates somatic mutations and gene expression in cancer. Correlations can be analyzed in two ways- The ‘Genotype’ run is for finding changes in gene expression that are related to a specific mutation and the ‘Target’ run is for finding mutations that alter the expression of target genes. The name of the candidate gene *MTUS1* was used as input and the specific cancers associated with the significant nsSNPs were selected [34].

## Result

**Fig 1:**
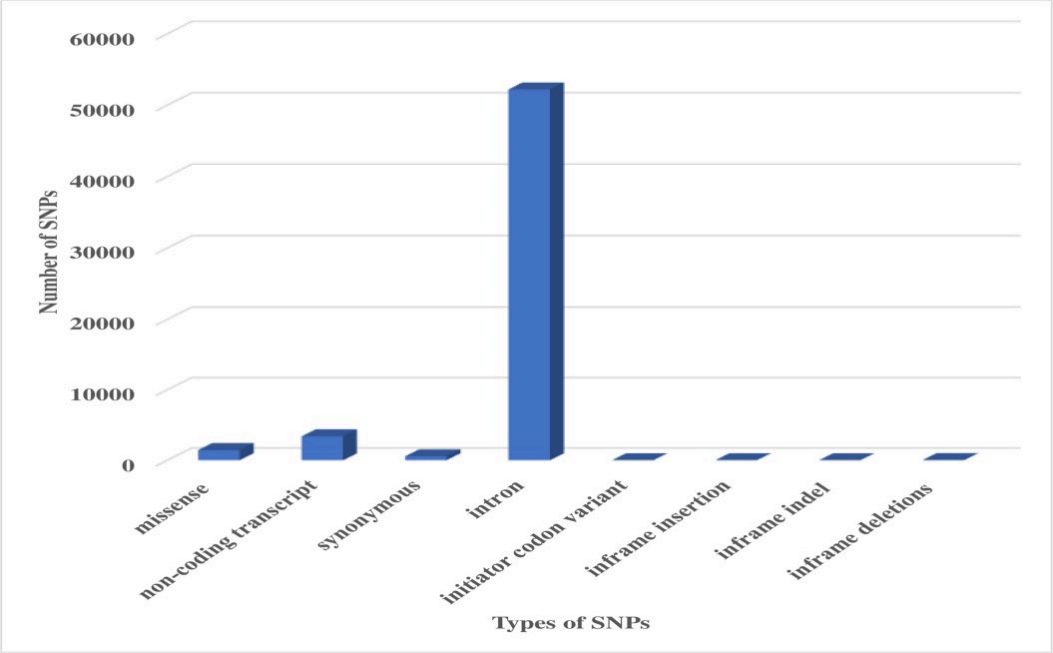
Distribution of SNPs in different functional classes of *MTUS1* gene obtained from the dbSNP database.

SNPs of the human *MTUS1* gene were retrieved from dbSNP database (dbSNP NCBI: https://www.ncbi.nlm.nih.gov/snp/?term=chek2). It comprised a total of 55120 SNPs, out of which 1453 were missense (nsSNP), 3400 non-coding transcript, 558 synonymous, 52194 intronic, 9 initiator codon variant, 1 inframe insertion, 4 inframe indel and 20 inframe deletions (Fig 1). For this study only nsSNPs of *MTUS1* were selected, which contributed to only 2.63% of all SNPs known in human *MTUS1* gene.

### Screening of functional nsSNPs in *MTUS1*

Single nucleotide variants of the *MTUS1* attained from dbSNP analysis were subjected to *in silico* analysis through variety of tools such as SIFT, SNAP2, Align GVGD, Polyphen-2, PROVEAN, PANTHER and PhD-SNP.

For initial screening we used SIFT that predicted a total 215 nsSNPs as tolerated or deleterious out of 1453 whereas the rest of nsSNPs were not found. Among these 215 nsSNPs, SIFT classified 108 nsSNPs as deleterious, 107 as tolerated (S1 Table). To filter the SIFT result, we performed SNAP2, Align GVGD, PolyPhen-2, PROVEAN, PANTHER and PhD-SNP. According to SNAP2, 104 variants (48%) were significant while the rest (52%) showed no effect (S1 Table). Whereas Align GVGD predicted 194 SNPs as most likely affected and 21 nsSNPs as less likely affected out of 215 nsSNPs (S1 Table).

**Fig 2:**
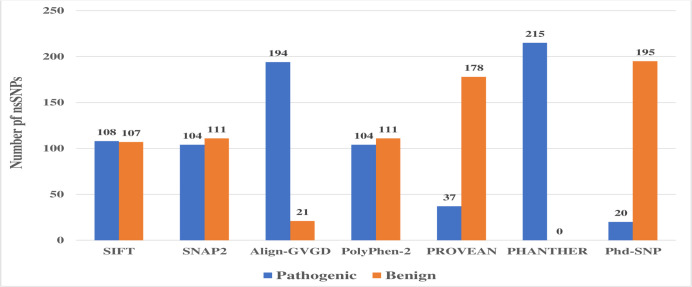
Prediction of pathogenicity of nsSNPs by SIFT, SNAP2, Align- GVGD, PolyPhen-2, PROVEAN, PANTHER and PhD-SNP software.

PolyPhen-2 speculated 67(31%) as probably damaging, 37(17%) as possibly damaging and the remaining (52%) as benign (S1 Table). PROVEAN suggested that, 178 amino acid substitutions (83%) were neutral (score is above-2.5) and the remaining 37 (17%) were diseases associated (score below or equal -2.5) (S1 Table). Interestingly all of the nsSNPs (215) were found to be probably damaging by PANTHER (S1 Table). In addition, out of the 215 nsSNPs of *MTUS1* gene, PhD-SNP revealed only 20 (9%) nsSNPs as diseased and rest of 195 (81%) nsSNPs as neutral (S1 Table) (Fig 2).

By using 7 Prediction tools (SIFT, SNAP2, Align GVGD, Polyphen-2, PROVEAN, PANTHER and PhD-SNP), eventually 9 significant nsSNPs (E934V, C264Y, S1259L, E960K, L1084V, P503T, H1077L, S1245Y and L1143Q) were selected as most deleterious (Table 1). To investigate the impact of these nsSNPs on the structure and function of the MTUS1 protein additional analysis were performed.

**Table 1:** Identification of deleterious nsSNPs by 7 *in silico* programs.

RsIDs	Amino Acid Change	SIFT	SNAP2	Align- GVGD	PolyPhen-2	PROVEAN	PHANTHER	Phd-SNP
rs138534724	E934V	Deleterious	Effect	C65	Probably Damaging	Deleterious	Probably damaging	Disease
rs138713013	C264Y	Deleterious	Effect	C65	Probably Damaging	Deleterious	Probably damaging	Disease
rs148435996	S1259L	Deleterious	Effect	C65	Probably Damaging	Deleterious	Probably damaging	Disease
rs181040560	E960K	Deleterious	Effect	C55	Probably Damaging	Deleterious	Probably damaging	Disease
rs181719146	L1084V	Deleterious	Effect	C25	Probably Damaging	Deleterious	Probably damaging	Disease
rs201397082	P503T	Deleterious	Effect	C35	Probably Damaging	Deleterious	Probably damaging	Disease
rs201647662	H1077L	Deleterious	Effect	C65	Probably Damaging	Deleterious	Probably damaging	Disease
rs370363143	S1245Y	Deleterious	Effect	C65	Probably Damaging	Deleterious	Probably damaging	Disease
rs373021974	L1143Q	Deleterious	Effect	C65	Probably Damaging	Deleterious	Probably damaging	Disease

### Analysis of protein structural stability

The selected 9 nsSNPs were analyzed by I-mutant Suite to reveal the effect of point mutation on protein stability based on free energy change value. Out of 9, 6 variants (C264Y, S1259L, E960K, L1084V, P503T and L1143Q) were predicted to decrease stability whereas others (E934V, H1077L and S1245Y) were found to increase protein stability (Table 2).

**Table 2:** Analysis of protein stability and evolutionary conservation profile of high risk nsSNPs of *MTUS1* by I-Mutant and ConSurf.

Amino Acid Change	I-Mutant		ConSurf		
	DDG Value Kcal /mol	Stability	Conservation Score	Buried/Exposed	Functional/Structrual
E934V	0.35	Increase	9	Exposed	Functional
C264Y	-0.45	Decrease	1	Exposed	-
**S1259L**	**-0.19**	**Decrease**	**9**	**Exposed**	**Functional**
**E960K**	**-0.65**	**Decrease**	**9**	**Exposed**	**Functional**
**L1084V**	**-1.17**	**Decrease**	**9**	**Buried**	**Structural**
**P503T**	**-1.41**	**Decrease**	**9**	**Exposed**	**Functional**
H1077L	0.52	Increase	7	Exposed	-
S1245Y	-0.21	Increase	9	Exposed	Functional
**L1143Q**	**-1.71**	**Decrease**	**9**	**Buried**	**Structural**

### Conservation profile of deleterious nsSNPs in *MTUS1*

Through the ConSurf web server, all the 9nsSNPs were analyzed to evaluate evolutionary conservation and find putative structural and functional residues. According to ConSurf output, out of 9nsSNPs 7 variants (E934V, S1259L, E960K, L1084V, P503T, S1245Yand L1143Q) were highly conserved residues (conservation score of 9) and remaining H1077L and C264Y were predicted moderately conserved (conservation score of 7) and variable (conservation score of 1) residue respectively. Among these 7 highly conserved residues, 5 variants (E934V, S1259L, E960K, P503T and S1245Y) were predicted as functional and exposed whereas remaining 2 (L1084V and L1143Q) were structural and buried. The result of ConSurf is shown in Table 2. The summary of deleterious prediction for each SNP by ConSurf is shown in S1–S3 Figs.

For the identification of the most deleterious nsSNPs, I-mutant and ConSurf output were compared and scrutinized. 5nsSNPs (S1259L, E960K and P503T, L1084V and L1143Q) were selected as potentially damaging based on this comparison which were subjected to further analysis.

### Prediction of solvent accessibility

Solvent accessibility and stability were assessed for the 5 variants (S1259L, E960K, P503T, L1084V and L1143Q) by NetsurfP. Among these deleterious nsSNPs, 3 variants (S1259L, E960K P503T) and their respective wild variants were exposed to the surface whereas 2 variants (L1084V and L1143Q) and their respective wild variants were buried (Table 3).

**Table 3:** Prediction of surface accessibility by NetSurfP.

Amino Acid Change	NetSurfP-2.0		
	Class Assignment	Relative surface Accessibility (RSA)	Absolute surface Accessibility (ASA) Å
S1259L	Exposed	0.833932	97.73682
E960K	Exposed	0.484737	84.68355
L1084V	Buried	0.184048	33.69914
P503T	Exposed	0.394134	55.92756
L1143Q	Buried	0.163323	29.90438

### The structural impacts of functional *MTUS1* mutations

**Fig 3:**
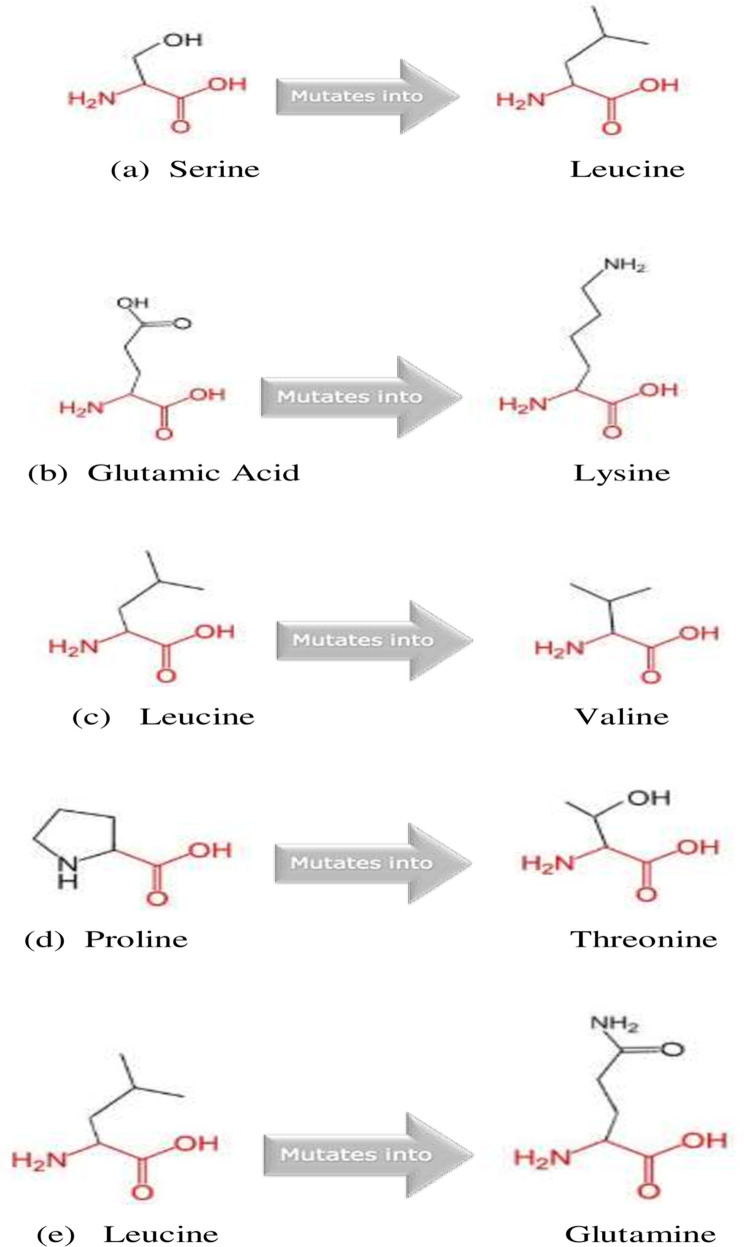
Schematic structures of the original (left) and the mutant (right) amino acid.

HOPE speculated the effect of amino acid substitution on the physical and chemical properties, hydrophobicity, spatial structure, and function of protein (Table 4). Based on HOPE, the mutant residue S1259L, E960K and L1143Q were bigger than the wild type whereas the mutant residue L1084V was smaller than wild type. In addition, the mutant S1259L increased hydrophobicity whereas the mutant E960K and L1143Q decreased hydrophobicity. And the differences of the hydrophobicity and size between wild-type and mutant residue can disrupt the H-bonding interactions with the neighboring residues, and hence the protein framework. Moreover, for the mutant residue P503T, the substitution of proline might harshly disrupt a special conformation of protein as well as decrease hydrophobicity (Fig 3).

The backbone, which is the same for each amino acid, is colored red. The side chain, unique for each amino acid, is colored black; (a) S1259L (b) E960K (c) L1084V (d) P503T (e) L1143Q.

**Table 4:** Interpretation of the impact of amino acid change on MTUS1 protein structure and stability by HOPE.

Amino Acid Change	Change of size	Change of Charge	Change of Hydrophobicity	Interpretation
S1259L	W<M		Increase	Due to the preference of another secondary structure, the mutant residue can slightly destabilize the local conformation of protein. This mutant residue is located near a highly conserved position and it is probably damaging to the protein. As well as it is bigger this might lead to bumps. Moreover, this mutation introduces a more hydrophobic residue at this position which can result in loss of hydrogen bonds and/or disturb correct folding.
E960K	W<M	Negative > Positive		The mutant residue is located near a highly conserved position. It introduces a residue with a charge opposite to the wild-type. This change can cause repulsion with other residues in the protein or ligands. Moreover, this mutant residue is bigger which might lead to bumps.
L1084V	W>M			The mutation does not prefer α-helices as secondary structure whereas wild-type prefers α-helix. The mutant residue is located near a highly conserved position and smaller, this might lead to loss of interactions.
P503T			Decrease	The wild-type residue is a proline which is known to be very rigid and therefore induce a special backbone conformation which might be required at this position. The mutation can disturb this special conformation and it is located near a highly conserved position. Furthermore, hydrophobic interactions, either in the core of the protein or on the surface, will be lost.
L1143Q	W<M		Decrease	The mutation does not prefer α-helices as secondary structure whereas wild-type prefers α-helix. This mutation is located near a highly conserved position and is probably damaging to the protein. Furthermore, hydrophobic interactions, either in the core of the protein or on the surface, will be lost.

### Analysis of protein-protein interaction

**Fig 4:**
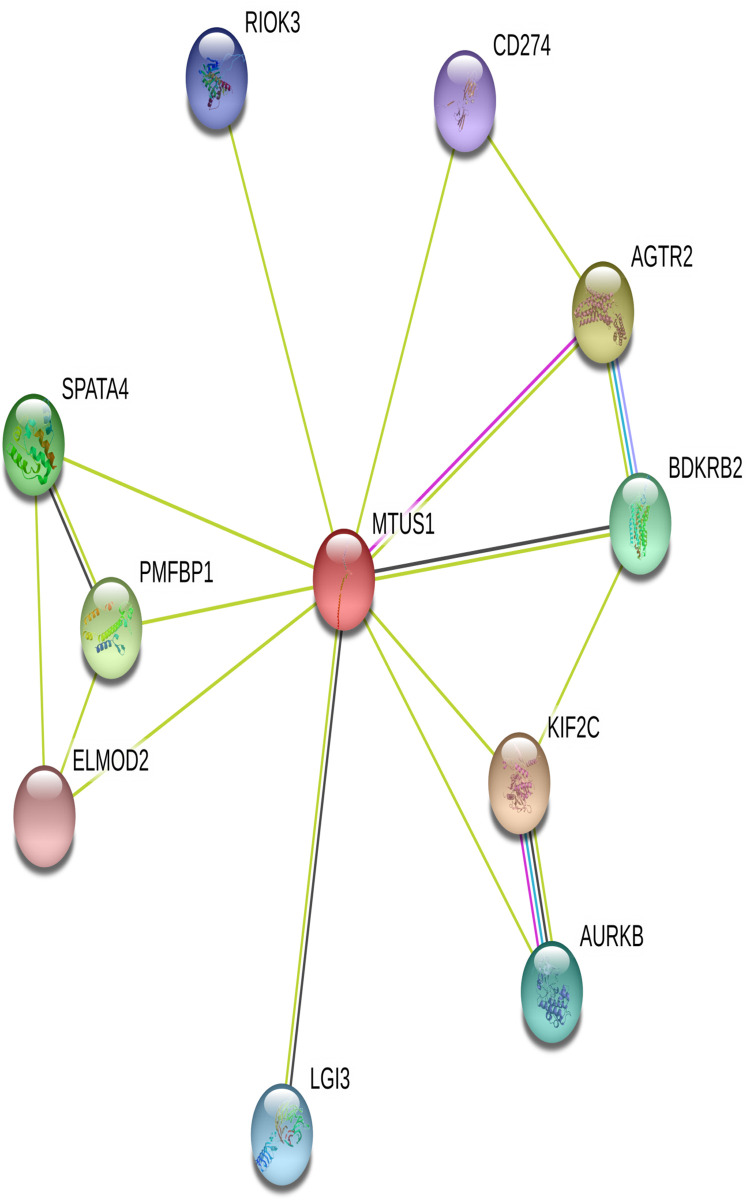
Protein–protein interaction network of MTUS1 using STRING server.

STRING revealed that products of *MTUS1* interacts with Angiotensin II Receptor Type 2(*AGTR2*), *CD274*, also commonly referred to as PDL1, Bradykinin Receptor B2 (*BDKRB2*), Kinesin Family Member 2C(*KIF2C*), Aurora kinases (*AURKB*), Leucine Rich Repeat LGI Family Member 3 (LGI3), ELMO Domain Containing 2 (ELMOD2), Polyamine Modulated Factor 1 Binding Protein 1 (PMFBP1), Spermatogenesis Associated 4 (SPATA4), and RIO Kinase 3 (RIOK3) (Fig 4).

### 3D structure of MTUS1 protein

The three-dimensional structure of the human MTUS1 protein was modeled using I-TASSER. The top 10 structural analogs in PDB were used as templates for such modeling, from which the topmost template (PDB ID: 6w1sr) covered 93% of the human MTUS1 query sequence. The server provided the top 5 models for the targeted protein though none of them were validated according to the scores of structure validation software due to their poor stereochemical properties (S2 Table).

**Fig 5:**
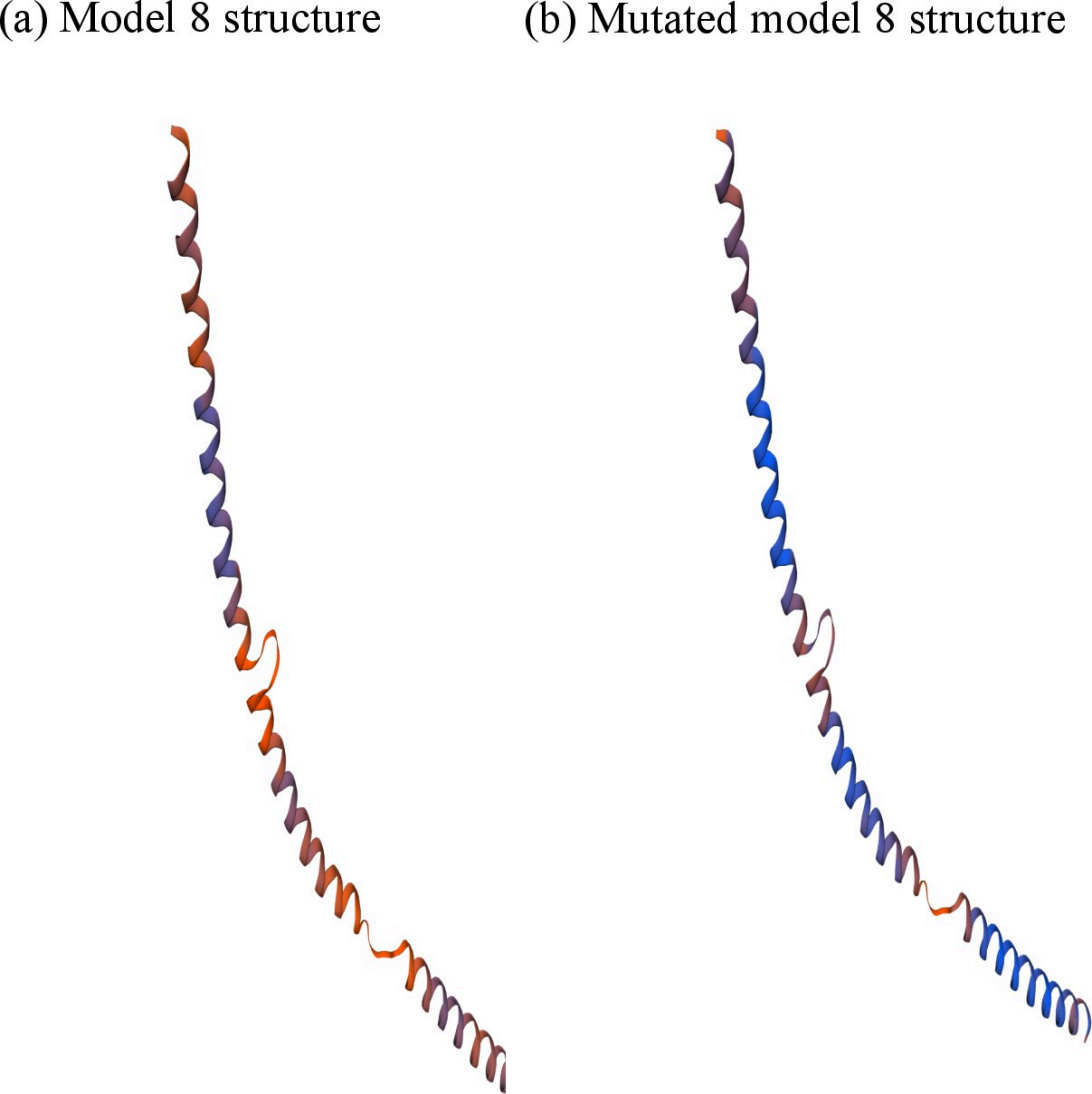
Homology models from SWISS-MODEL server; (a) Structure of Model-8 (b) Mutated Model-8 structure.

Hence, we used SWISS-MODEL for the three-dimensional structural analysis of MTUS1 protein. Unlike I-TASSER, SWISS-MODEL provided 9 structures and all of them were a partial structure of our targeted protein based on the best-aligned template from UniProtKB proteome. Further analyses were done using PROCHECK, QMEAN, Molprobity, ERRAT, and ProSA programs to estimate the quality of the models (Table 5), and model number 8 was selected as the best one (Fig 5).

**Table 5:** Scores of different structural assessment tools for the predicted models from SWISS-MODEL homology-modeling server.

Model number	SWISS-MODEL Ramachandran Favorable region (%)	PROCHECK Core region of Ramachandran Plot (%)	QMEAN Score	Molprobity Score	ERRAT Score	PROSA Z- score
01	95.96	95.4	1.44	1.96	97.8378	-1.25
02	96.48	95.7	0.09	1.72	92.1348	-1.14
03	94.06	90.2	-1.51	1.88	79.8046	-2.01
04	89.96	88.2	-2.67	1.92	97.2332	-3.38
05	93.98	93.9	1.07	1.56	93.4653	-1.11
06	94.26	92.8	-0.66	1.43	89.2035	-1.27
07	93.06	91.6	-1.81	1.92	94.6565	-2.88
**08**	**98.04**	**95.1**	**1.08**	**0.82**	**96.875**	**-0.83**
09	95.71	94.2	-1.56	1.96	91.3706	-1.02
Mutated Model	97.06	94.1	1.08	0.86	96.87	-

For model 8- Ramachandran favored region was 98.04% by SWISS-MODEL and 95.1% core region by PROCHECK analysis. Molprobity and QMEAN scores were 1.08 and 0.82 respectively. ERRAT showed a higher score of 96.875% and ProSA-Z score was -0.83 for the best-predicted model.

### Structure and comparison of the mutated protein

In our study, the most suitable model (model number 8) covered the amino acid residues from 1133–1236 and it contained only one mutation (L1143Q) out of the investigated 5 most deleterious nsSNPs. Instead of Leucine, Glutamine was introduced at the position of 1143 in the native protein sequence and SWISS-MODEL was used to determine the mutated structure. While verifying the mutated model quality by SWISS-MODEL structure assessment, PROCHECK, QMEAN, Molprobity, and ERRAT similar scores were found as its native structure (Table 5).

TM-align, an algorithm for protein structure alignment and comparison was used for comparing the structural analog. For mutated structure, we got 0.99982 and 0.05 as TM-score and RMSD value respectively. These values indicate that both the structures are in the same fold.

### Association of the damaging nsSNPs with cancer

The mutation profile of *MTUS1* in both cBioPortal and canSAR Black webserver revealed the association of S1259L with Stomach adenocarcinoma (STAD) and the substitution of glutamate at 960 position with Uterine corpus endometrial carcinoma (UCEC). The canSAR black webserver also revealed that S1259L may result in moderate STAD whereas mutation at 960 may result in severe UCEC. However, the remaining nsSNPs were not found in the mutation profile presented by the web servers.

### Expression analysis of *MTUS1* gene

**Fig 6:**
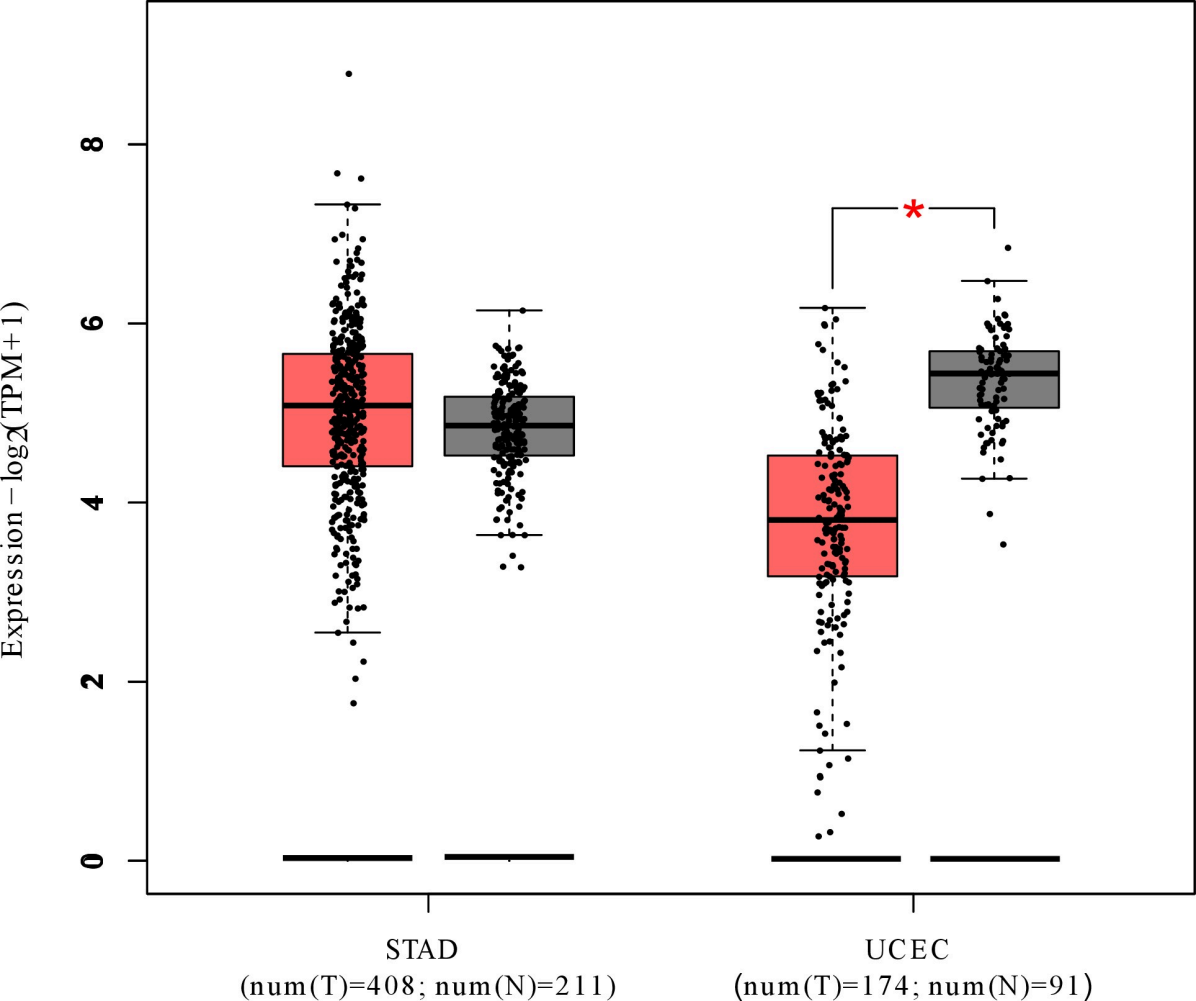
Box plot analysis of *MTUS1* gene expression in case of STAD and UCEC for both tumor and normal samples.

The box plot analysis revealed that, Stomach adenocarcinoma (STAD) results due to overexpression of *MTUS1* whereas Uterine corpus endometrial carcinoma (UCEC) ensues due to underexpression of *MTUS1* (Fig 6).

### Survival analysis in STAD and UCEC patients

**Fig 7:**
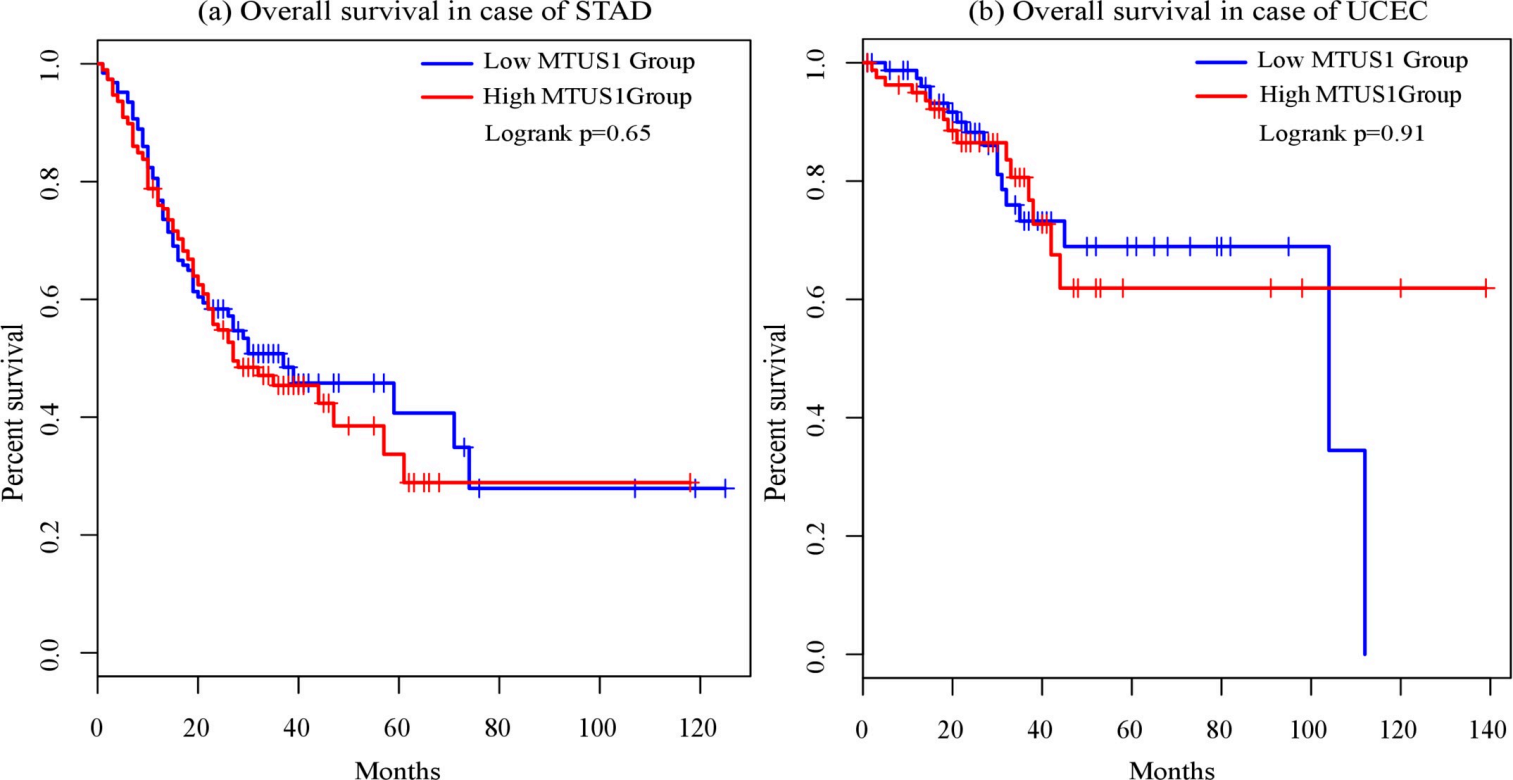
Overall survival (in percent) for both STAD and UCEC.

We used the GEPIA database for survival analysis of patients with Stomach adenocarcinoma (STAD) and Uterine corpus endometrial carcinoma (UCEC). The patients were categorized into high-expression and low-expression groups based on the median expression level of *MTUS1*. Abnormal expression of *MTUS1* displayed association with poor prognosis of patients with STAD and UCEC. In STAD, overexpression of *MTUS1* showed shorter survival time compared to patients with lower expression levels. In contrast, UCEC patients with high *MTUS1* expression had longer survival time than patients with lower *MTUS1* expression (Fig 7).

### Relationship between mutation and expression of *MTUS1* gene with other significant genes

**Fig 8:**
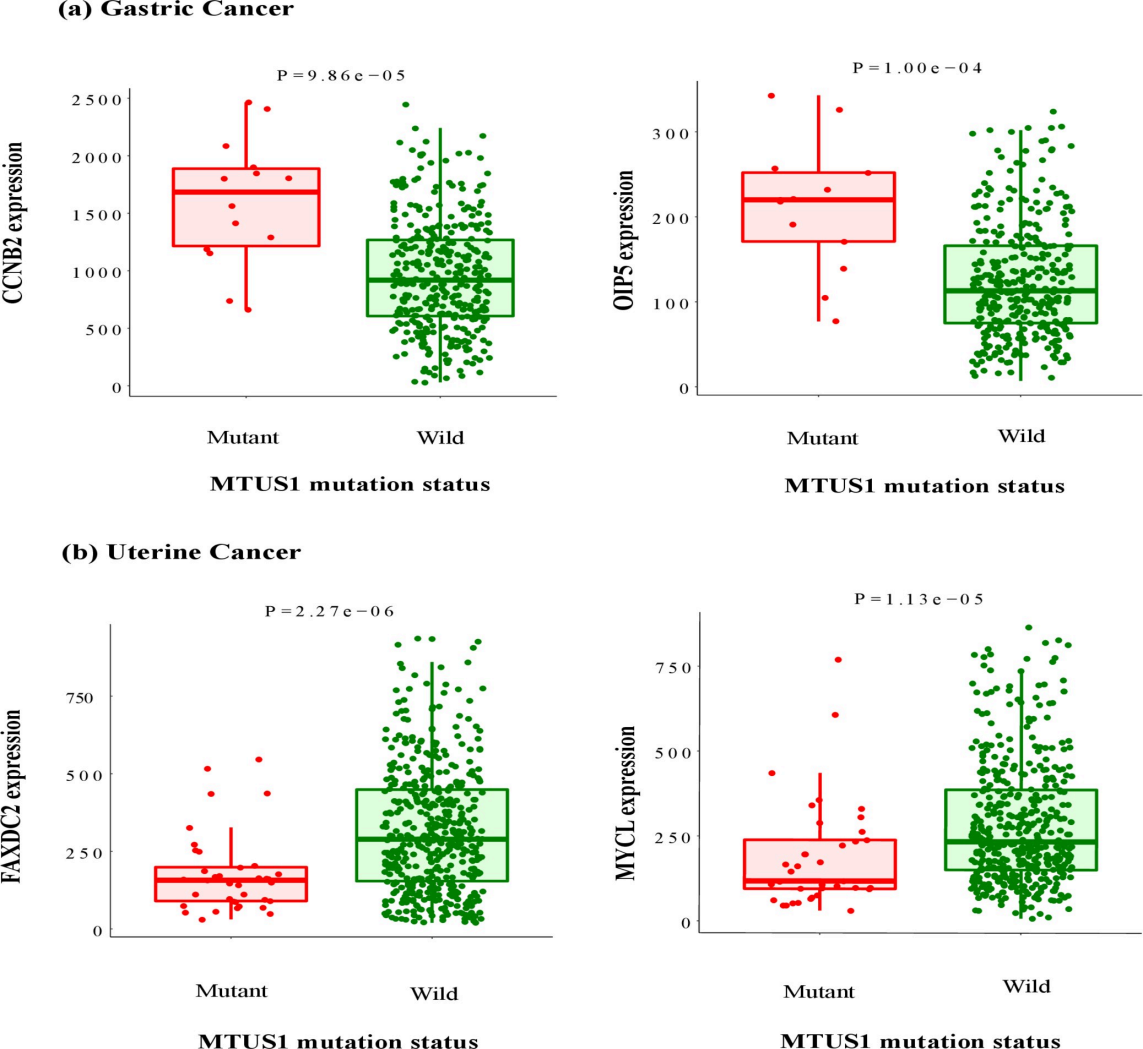
Genotype run for the changes in *MTUS1* gene expression; (a) Gastric Cancer (b) Uterine cancer.

**Fig 9:**
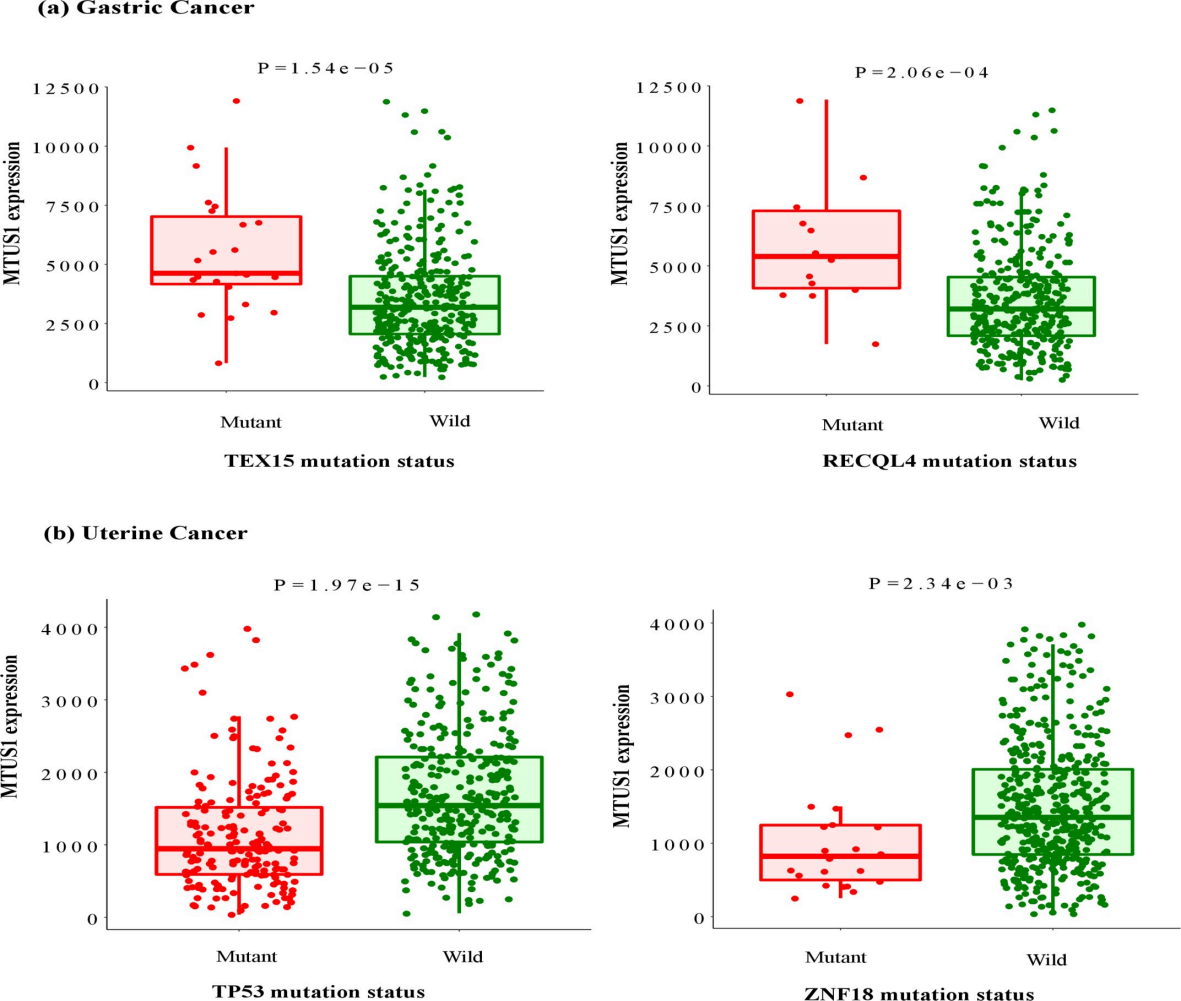
Target run for mutation in significant genes that alter the *MTUS1* expression; (a) Gastric Cancer (b) Uterine cancer.

The muTarget links mutation with gene expression alteration and provides expression plots. We used it for two independent analysis. Firstly, the ‘Genotype’ run with *MTUS1* as mutated gene showed that for Gastric cancer, mutation in *MTUS1* can affect the expression of other genes such as *CCNB2*, *OIP5* etc. This same analysis for Uterine cancer showed that expression of *FAXDC2*, *MYCL* etc. altered due to *MTUS1* mutation (Fig 8). Secondly, the ‘Target’ run with *MTUS1* as the target gene represented that altered genes of Gastric cancer (i. e., *TEX15*, *RECQL4*) can change expression of *MTUS1*. Similarly, expression of *MTUS1* can be changed due to the mutation of genes responsible for Uterine cancer (i.e., *TP53*, *ZNF18*). Though the server provides up to top 30 models, we represented expression of two genes for each analysis (Fig 9).

## Discussion

Mitochondrial tumor suppressor 1 (*MTUS1*) is a tumor suppressor gene which has been associated with various cancers i.e. breast, colon, lung, pancreas, bladder and prostate cancer [3,4]. The gene encodes a family of angiotensin II type 2 (AT2) receptor interacting proteins (ATIPs) which exert anti-tumor effects by inhibiting cell proliferation as well as differentiation [7].

In recent years presence of damaging SNPs in several oncogenes [35,36] has made *in silico* investigation of deleterious SNPs from large datasets a major concern. Despite the impact of *MTUS1* has been annotated in the cell proliferation of several carcinomas, the *in silico* analysis of deleterious nsSNPs in our candidate *MTUS1* gene has remained uncharacterized yet.

The effectiveness of the most deleterious SNPs prediction can be made more valid by combining several computational based approaches together. In this study, we performed the analysis with 7 prediction tools (SIFT, SNAP2, Align-GVGD, Polyphen-2, PROVEAN, PANTHER and PhD-SNP) to get an integrated picture of pathogenic SNPs of *MTUS1* gene. Owing to the reliance of each algorithm on different parameters, in this study we selected 9 nsSNPs (Table 1) as high-risk that were predicted to be deleterious by all SNP prediction algorithms for further analysis.

Generally, the function, activity and regulation of a protein significantly depend on the structural stability of the molecule. Decrease in protein stability causes degradation, misfolding and aggregation of proteins leading to subsequent dysfunction [37,38]. To determine the effect of the above mentioned 9 deleterious nsSNPs on the stability of the MTUS1 protein, I-mutant suite was used. Out of these 9 nsSNPs, 6 decreased the stability of the protein hence may have impact on protein dysfunction.

The evolutionary conservation profile of a protein helps to determine the severity of a detrimental mutation. The nsSNPs that are located at highly conserved region are more likely to be detrimental than nsSNPs that are located at variable regions [39]. We used ConSurf web server to inspect potential effects of these 9 most deleterious nsSNPs (Table 2). For finding putative structural and functional sites, ConSurf offers evolutionary conservation data with solvent accessibility predictions [21]. Furthermore, based on their position relative to the protein surface or protein core, highly conserved residues are subjected to be either structural or functional respectively [40]. According to ConSurf, 7 deleterious nsSNPs among 9 nsSNPs had high conservation score and out of these 7 highly conserved nsSNPs, 5 were predicted as functional (exposed) whereas the rest of them were predicted to be structural (buried).

By analyzing the result of I mutant and ConSurf, we can conclude that out of these 9 nsSNPs, 5nsSNPs (S1259L, E960K, P503T, L1084V and L1143Q) are potentially vulnerable because of their higher conservancy as well as ability to decrease protein stability.

Moreover, to analyze the effect of these above-mentioned 5 high-risk nsSNPs on the structure of MTUS1 protein we performed NetsurfP and HOPE. NetsurfP speculates solvent accessibility and secondary structure of the protein [22]. It presented 3 variants as exposed (S1259L, E960K and P503T) and other 2 variants as buried (L1084V and L1143Q), which can be verified by the similar findings from ConSurf web server.

Determination of protein stability is quite difficult as it cannot be predicted by considering a single factor only. Physiochemical properties of amino acid residues such as-polarity, charge, hydrophobicity etc. play crucial role for the overall stability of the protein molecule. It has been shown that protein stability is linked with the presence of hydrophobic residues at its surface area because these residues hinder accessibility of water molecule to the protein [41]. Among the exposed variants, in case of S1259L, the exposure of the non-polar residue (Leucine) in place of the polar one (Serine) on the surface area may decrease protein stability. Again, in E960K the substitution of a negatively charged molecule (Glutamic acid) with a positively charged one (Lysine) can cause repulsion which may severely hamper the interaction of the protein with other molecules. However, in P503T, a nonpolar group (Proline) is replaced by a polar group (Threonine) which may decrease the hydrophobicity of surface area. Between the 2 buried variants, in case of L1084V there is no polarity change, however in L1143Q, a nonpolar group (Leucine) is substituted by a polar group (Glutamine) which may decrease the hydrophobicity of the protein core. These findings can be further validated by HOPE result.

Hope server predicted that these 5 highly risky nsSNPs might have damaging effect on the structure of the protein (Table 4) among which, 2 nsSNPs were structural and 3 nsSNPs were functional residues according to ConSurf.

For the understanding of cellular processes protein–protein interaction network is a vital factor. STRING plays a critical role to filter and assess functional genomics data as well as to provide an intuitive platform for interpreting structural, functional and evolutionary properties of proteins [24]. In the present study, this database revealed the interaction of MTUS1 protein with other proteins which may involve in different pathways, and disruption of these pathways may result in diseases.

To determine the three-dimensional structure of human MTUS1 protein, structural prediction tools were used due to the unavailability of the PDB ID (Protein Data Bank ID) of this protein. Automated protein structure prediction tool, I-TASSER was used where FASTA sequence of the whole protein was given as an input file and the server came up with the top 5 final structural models. The quality of the predicted models is preliminarily estimated by the confidence score (C-score) given by the server that ranges from -5 to 2 and the highest score depicts the most compatible ones. According to this, the first model (highest C-score = -1.67) was selected as the most suitable structure [25]. As there is a possibility of having a better quality of low-ranking models than that of high-ranking in rare cases, hence, all the models were further analyzed for structural validation.

Validation of experimental models is indispensable to obtain a better quality of targeted protein structure. To ensure it several computational programs- SWISS-MODEL, PROCHECK, QMEAN, Molprobity, ERRAT, and ProSA were used. Among all the verification matrices Ramachandran plot is the most prioritized as it represents the φ-ψ torsion angles of the protein backbone of predicted models. SWISS-MODEL gives the idea about the favored region of the Ramachandran plot and PROCHECK reveals the stereochemical quality of a given protein structure by assessing the Ramachandran plot into different regions—core, allowed, generously allowed, and disallowed region. More than 90% of residues in the core region or in the most favored region can be preferred as a favorable structure [42].

Other computational tools provide scores for the estimation of protein model quality. QMEAN-Z score of -4.0 or below -4.0 indicates the model with low quality and higher score points to the favorable states of the structure [43]. ProSA-Z score of -4.0 or below depicts the poor quality of models like QMEAN [44]. Around 95% or higher value of ERRAT score generally provides high standard resolution of structure [42]. On the other hand, Molprobity score closer to zero represents the higher quality of a structure [45].

In our study, all the 5 models from I-TASSER were invalidated according to the standard score of validation tools mentioned above (S2 Table). However, we provided our query sequence to SWISS-MODEL, a homology-modeling server. Based on the best-aligned template, the server came up with 9 models, although none of them covered the whole sequence of our targeted protein. Interestingly, all of the 9 models fell into the range of amino acids from 906 to 1239 which might indicate that the protein sequence around this region could be a conserved region. Our findings from ConSurf analysis also showed a similar result. All of our predicted 5 significantly damaging nsSNPs (S1259L, E960K, L1084V, P503T, and L1143Q) were highly conserved and were in between this region.

All these 9 models were then evaluated for structural validation by PROCHECK, QMEAN, Molprobity, ERRAT, and ProSA programs. According to the standard score of all validation software, model number 8 was selected as the best presumptive structure of MTUS1 protein (Table 5). This model comprises 104 amino acid residues that range from 1133–1236 based on template alignment and only one mutation (L1143Q) out of the 5 most deleterious nsSNPs fell into this range. To model the mutant structure, a point mutation at this specific position was made in the native protein sequence and provided to SWISS-MODEL. Only one mutated model was provided by the server and it showed similar scores as model 8 while verified by PROCHECK, QMEAN, Molprobity, and ERRAT programs (Fig 5 and Table 5).

Structural comparison between wild type and mutant structure was analyzed by TM-align tool. Low TM score and high RMSD value indicate structural dissimilarity whereas we found a high TM-score (0.99982) and low RMSD value (0.05); indicate both the structures are on the same fold [38]. The wild-type structure is of a snippet of the whole structure and only one mutation that falls into that region might be the reason for showing the similarity between native and mutant structures.

Mutation of a protein leads to genomic instability which may result in various types of cancers. To investigate such correlations different cancer prognostic tools were used. The Cancer genomics database cBioPortal provided us with a summary of types of cancers associated with *MTUS1*. According to this study, more than 30 cancers can occur due to various anomalies (i. e., mutation, structural variant, amplification, deep deletion and multiple alterations) in *MTUS1* gene. Interestingly, mutation is the sole reason for several cancers and high frequency of mutation has been observed in Uterine corpus endometrial carcinoma (UCEC) and Stomach adenocarcinoma (STAD) which account 6.99% and 3.86% respectively (S4 Fig).

We meticulously searched both cBioPortal and canSAR Black webserver for the 5 deleterious nsSNPs in the Lollipop plot that showing mutation frequency along the MTUS1 amino acid sequence. Among these 5 nsSNPs, the mutation S1259L was found to be associated with Stomach adenocarcinoma (STAD) and substitution of glutamate at 960 position was linked to severe Uterine corpus endometrial carcinoma (UCEC).

GEPIA provided an expression dot plot for all caner types related to *MTUS1*(S5 Fig). This revealed that most of the cancers occurred due to the downregulation of *MTUS1* though some of them resulted from upregulation of *MTUS1*. To represent the opposite expression of *MTUS1* gene more clearly, we performed box plot analysis of *MTUS1* gene expression in STAD and UCEC where these cancers resulted from upregulation and downregulation of *MTUS1* accordingly (Fig 6).

The survival curve is a plot of the survival probability (percentage) against time which provides crucial summary of data that is utilized to calculate measures i.e., median survival time [33,46]. In this study, survival analysis revealed that STAD patients with high levels of *MTUS1* had shorter survival time whereas UCEC patients with reduced expression of *MTUS1* had decreased survival time. This finding is consistent with the results we found in *MTUS1* gene expression analysis. So, both upregulation [47] and downregulation of *MTUS1* can lead to cancer progression though the molecular mechanisms underlying this process is still uncharted and can be a new agenda of research.

The muTarget is a tool that is designed to find out not only the genes whose expression are changed due to mutation in the query gene but also those genes whose mutations affect the expression of the target gene. From our analysis, it has been found that the expression of various genes was changed due to mutation in *MTUS1*. For instance, in case of gastric cancer, the expression of *CCNB2*, *OIP5* etc. genes were found to be altered along with *MTUS1* mutation. The *CCNB2* gene is involved in cell cycle regulation [48] whereas *OIP5* gene is associated with chromosomal segregation during mitosis [49]. For Uterine cancer, *FAXDC2* and *MYCL* gene’s expression changed due to *MTUS1* mutation. The *FAXDC2* gene induces megakaryocyte differentiation [50] whereas *MYCL* encodes a transcription factor concerned in lung cancer [51].

Again, when *MTUS1* was used as the target gene, its expression was changed by numerous mutated genes. For instance, the server represented that mutation in *TEX15*, *RECQL4* etc. genes altered the expression of *MTUS1* in STAD patients. *TEX15* is engaged in normal chromosome synapsis and metabolic recombination during spermatogenesis [52] whereas *RECQL4* encodes a DNA- dependent ATPase which may regulate chromosome segregation [53]. The expression of our target gene changed in the UCEC patients likewise owing to the mutations in genes like *TP53* and *ZNF18*. Interestingly, *TP53* is a tumor suppressor gene [54] like our candidate *MTUS1* gene. This interaction indicates that aberration in one tumor suppressor may affect other gene of similar category which might worsen the state of the disease [55]. However, *ZNF18* is a zinc finger protein involved in transcriptional regulation [56]. Such gene-gene interactions can be used to identify cancer biomarkers along with therapeutic drug targets and different treatment options in various cancers.

Two nsSNPs from the five deleterious nsSNPs have been found to be associated with cancers. It is highly likely that the other three nsSNPs might be involved in different cancers. However, robust *in vivo* investigation is needed to establish the association with specific cancer in future.

## Conclusions

As a tumor suppressor, the *MTUS1* gene products play critical roles in various cellular mechanisms and prevent uncontrolled cell growth and proliferation. Consequently, alteration of this gene has been associated with different types of diseases including various cancers.

This study is the first systematic and extensive *in silico* analysis of functional SNPs in the *MTUS1* gene. We reported 5nsSNPs (S1259L, E960K, P503T, L1084V and L1143Q) as potentially damaging due to their presence in the highly conserved region and ability to affect protein stability. And two of them, S1259L and E960K were found to be associated with Stomach adenocarcinoma and Uterine corpus endometrial carcinoma accordingly. The findings of this study will hopefully provide a guideline in extricating the damaging SNPs which increase the risk of cancers and disease susceptibilities. However, extensive population-based studies along with clinical trials are essential to characterize the effects of these polymorphisms on structure and function of the protein as well as to develop effective and individualized treatment option.

## Supporting information

S1 Fig Conservation profile of *MTUS1* (Amino Acid 1–600). (TIF)

S2 Fig Conservation profile of *MTUS1* (Amino Acid 601–1200). (TIF)

S3 Fig Conservation profile of *MTUS1* (Amino Acid 1201–1270). (TIF)

S4 Fig Cancer types summary that are related to *MTUS1*. (TIF)

S5 Fig Profile of *MTUS1* from GEPIA for gene expression related to all cancer types. (TIF)

S1 Table Identification of the impact of nsSNPs of the *MTUS1* gene. (DOCX)

S2 Table Validation scores of different computational tools for the models from I-TASSER. (DOCX)
