# Supplementary figures and images for: In silico analysis of deleterious SNPs of human MTUS1 gene and their impacts on subsequent protein structure and function
Source: PLoS One. 2021 Jun 14;16(6):e0252932. doi: 10.1371/journal.pone.0252932 (PMC8202925; doi:10.1371/journal.pone.0252932)

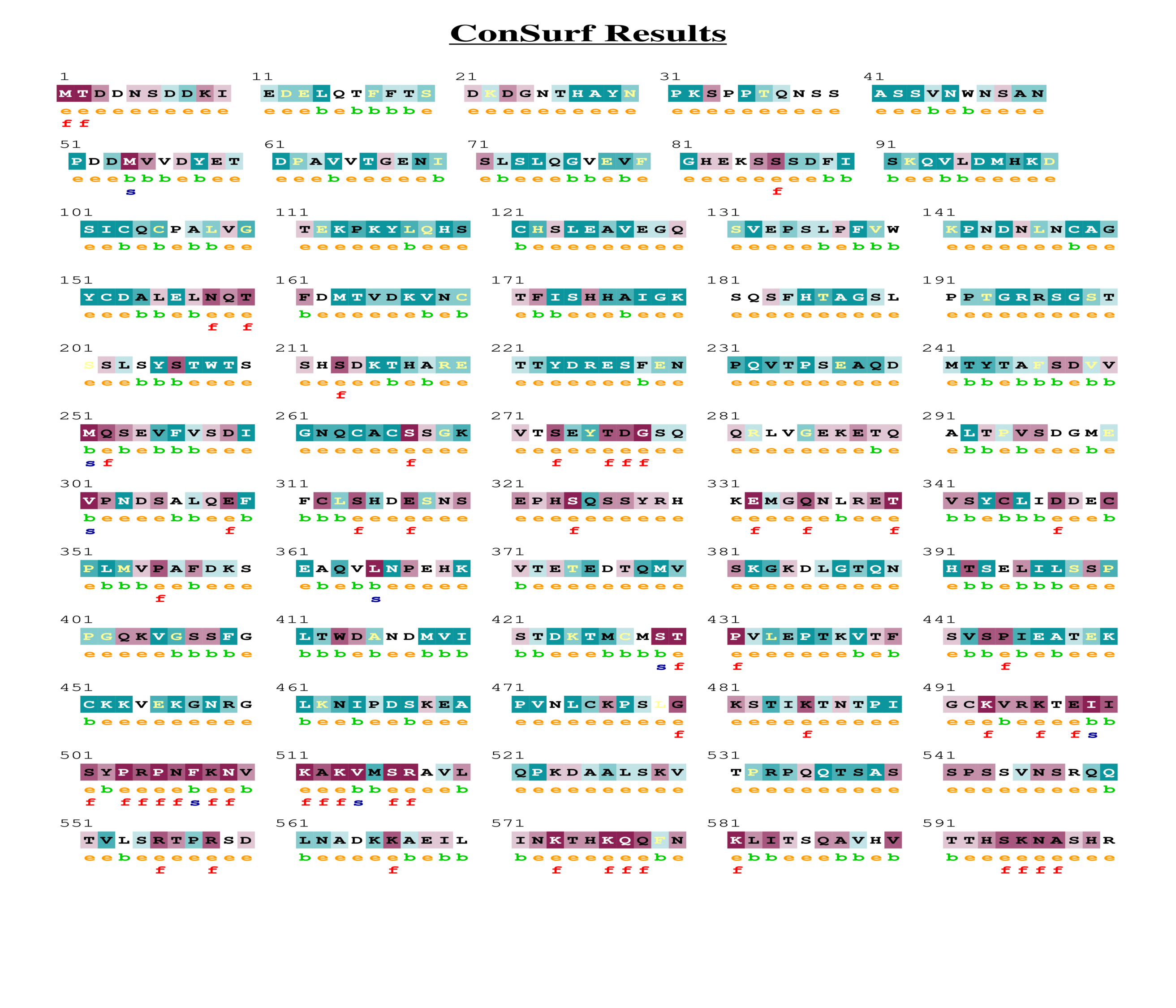

Supplement: S1 Fig — (TIF) [file pone.0252932.s001.tif]

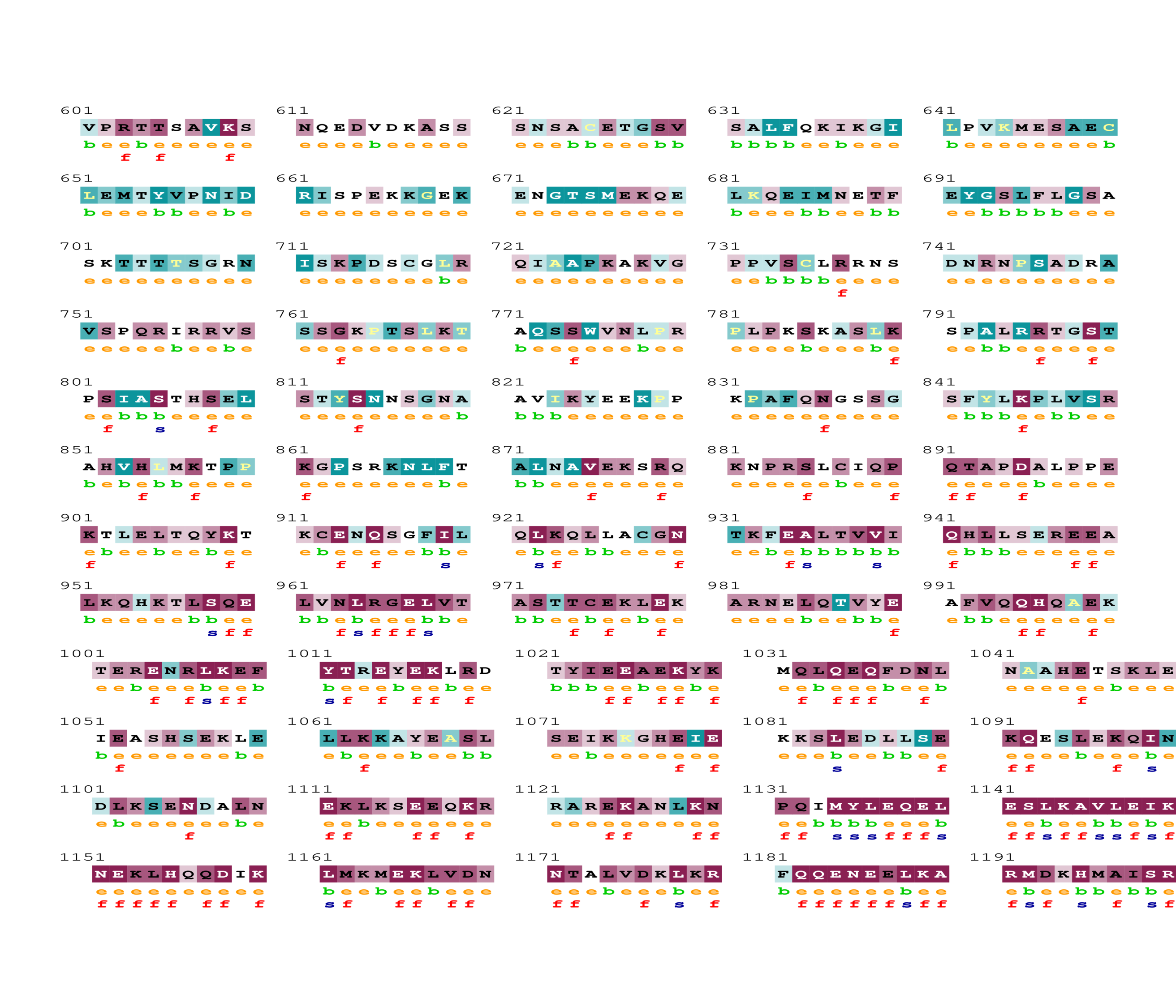

Supplement: S2 Fig — (TIF) [file pone.0252932.s002.tif]

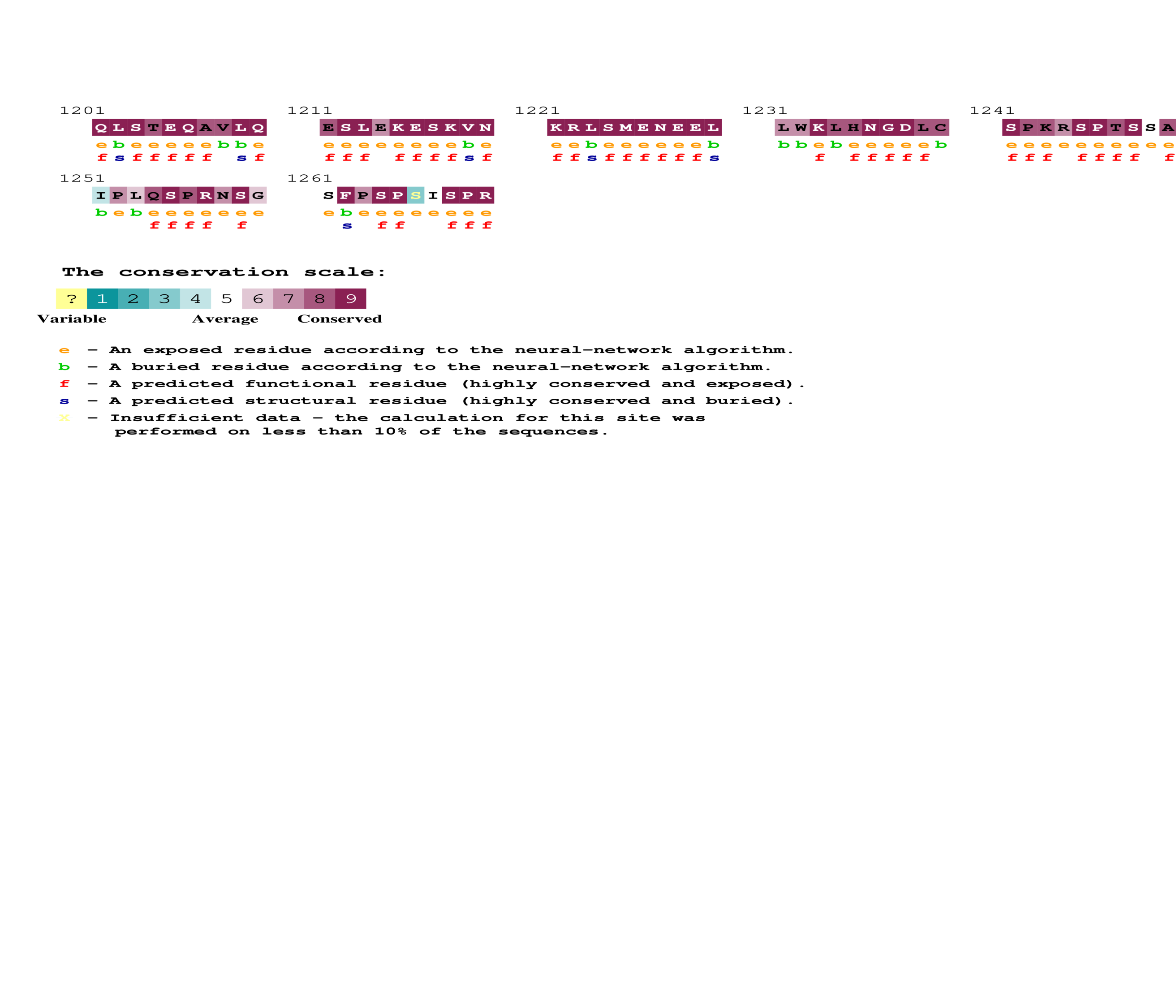

Supplement: S3 Fig — (TIF) [file pone.0252932.s003.tif]

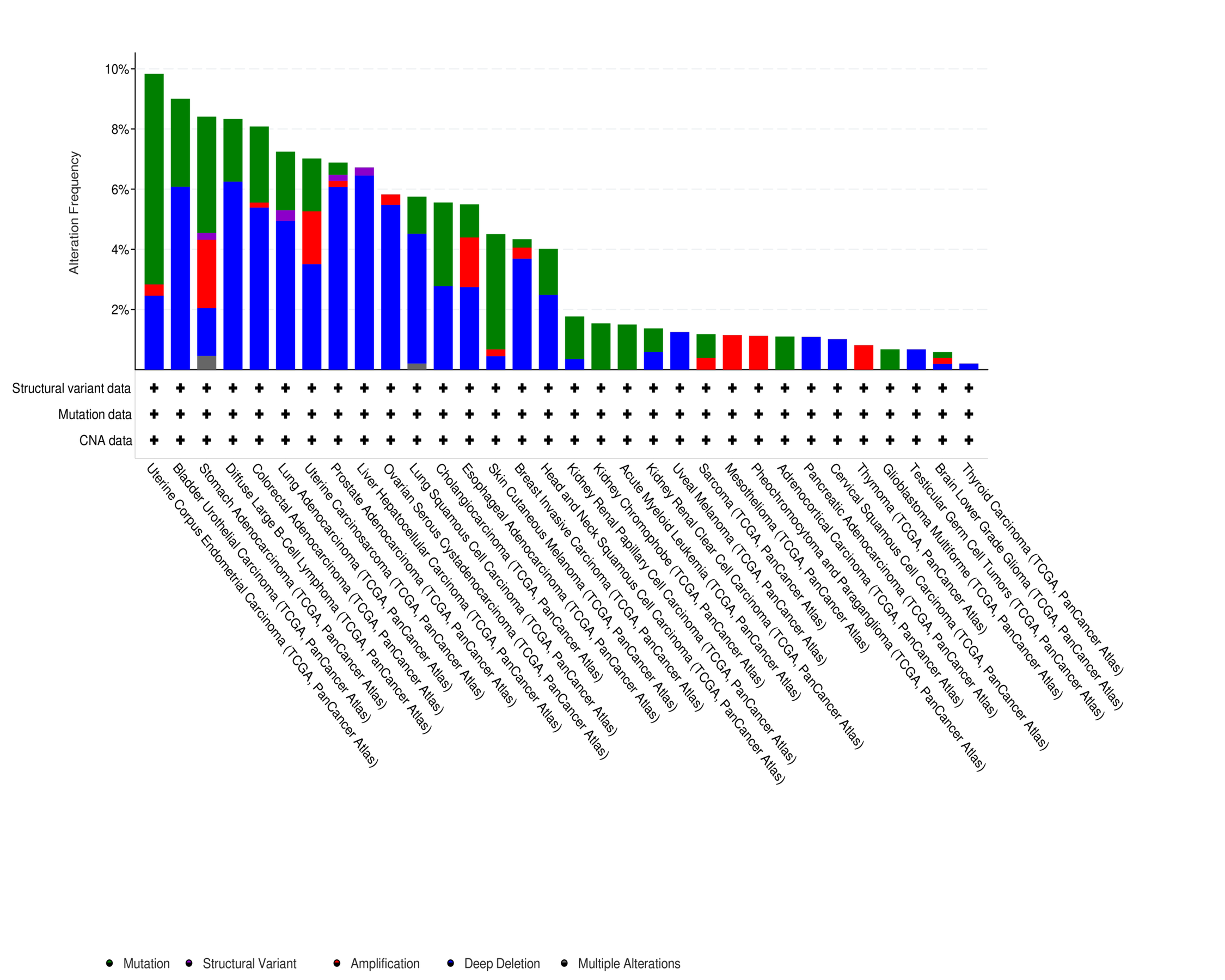

Supplement: S4 Fig — (TIF) [file pone.0252932.s004.tif]

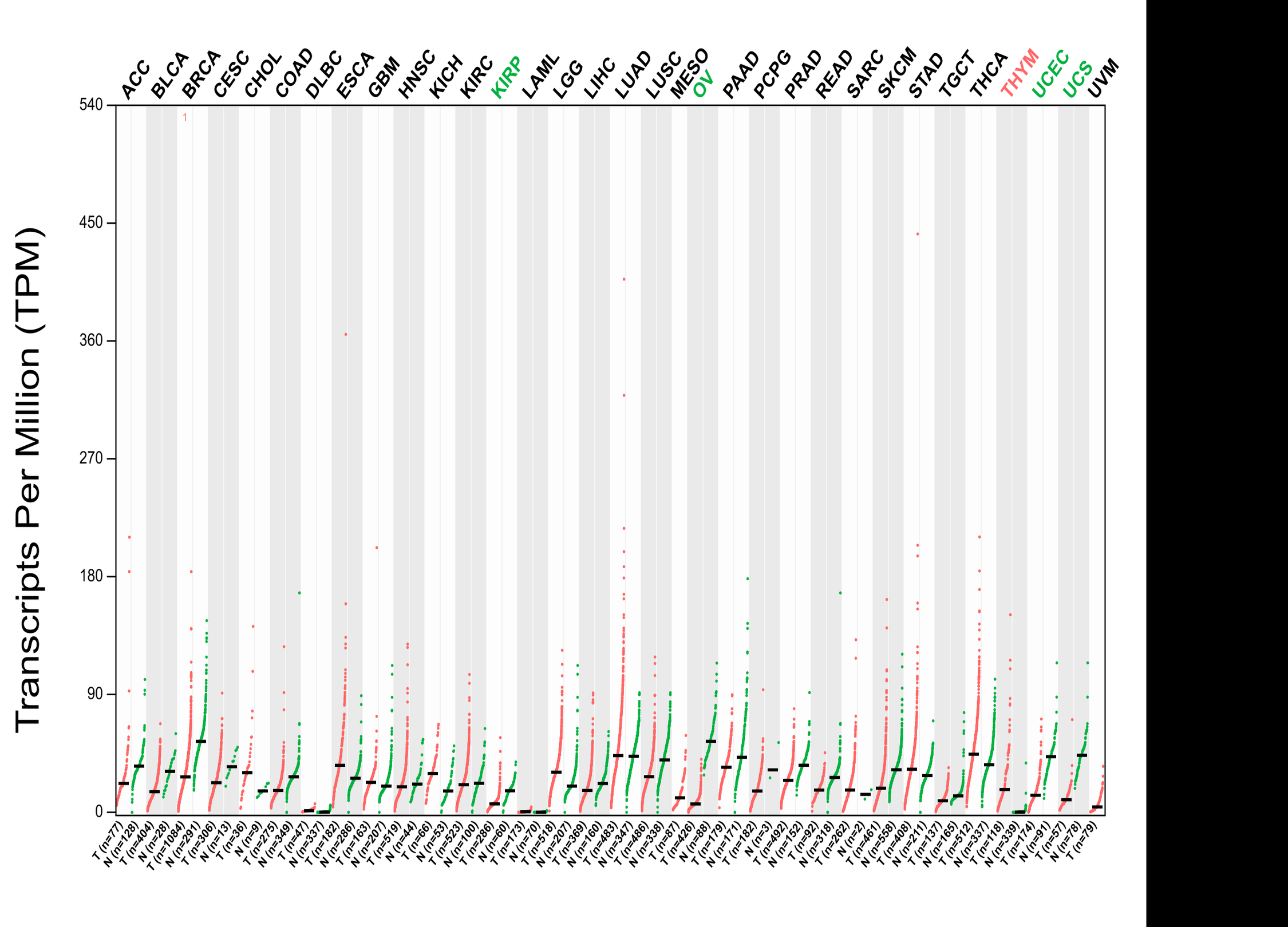

Supplement: S5 Fig — (TIF) [file pone.0252932.s005.tif]
