# Supplementary material for: In silico analysis of deleterious SNPs of human MTUS1 gene and their impacts on subsequent protein structure and function
Source: PLoS One. 2021 Jun 14;16(6):e0252932. doi: 10.1371/journal.pone.0252932 (PMC8202925; doi:10.1371/journal.pone.0252932)
Supplement: S1 Table — (DOCX) [file pone.0252932.s006.docx]

| **rsIDs** | **Amino Acid Change** | **SIFT** | **SNAP2** | **Align GVGD** | **PolyPhen-2** | **PROVEAN** | **PHANTHER** | **Phd-SNP** |
| --- | --- | --- | --- | --- | --- | --- | --- | --- |
| rs209569 | H575R | Deleterious | Effect | C25 | Possibly Damaging | Neutral | Probably damaging | Neutral |
| rs61733703 | Q75K | Deleterious | Effect | C45 | Possibly Damaging | Neutral | Probably damaging | Neutral |
| rs61733708 | E999G | Deleterious | Effect | C65 | Possibly Damaging | Deleterious | Probably damaging | Neutral |
| rs75253845 | M418R | Tolerated | Effect | C65 | Benign | Neutral | Probably damaging | Neutral |
| rs3739407 | C148R | Deleterious | Effect | C65 | Possibly Damaging | Neutral | Probably damaging | Disease |
| rs17690844 | K453T | Tolerated | Neutral | C65 | Benign | Neutral | Probably damaging | Neutral |
| rs17853231 | K1063T | Deleterious | Effect | C65 | Benign | Deleterious | Probably damaging | Neutral |
| rs61733690 | H854N | Deleterious | Neutral | C65 | Probably Damaging | Deleterious | Probably damaging | Neutral |
| rs61733691 | E646Q | Deleterious | Effect | C25 | Probably Damaging | Neutral | Probably damaging | Neutral |
| rs61733694 | T425M | Tolerated | Effect | C65 | Benign | Neutral | Probably damaging | Neutral |
| rs61733696 | S267P | Deleterious | Effect | C65 | Probably Damaging | Neutral | Probably damaging | Neutral |
| rs61733697 | S211A | Deleterious | Neutral | C65 | Benign | Neutral | Probably damaging | Neutral |
| rs61733698 | T207A | Tolerated | Neutral | C55 | Benign | Neutral | Probably damaging | Neutral |
| rs61733701 | A149T | Tolerated | Neutral | C55 | Benign | Neutral | Probably damaging | Neutral |
| rs61733702 | C103S | Deleterious | Effect | C65 | Benign | Neutral | Probably damaging | Neutral |
| rs61733705 | E1105Q | Tolerated | Effect | C25 | Possibly Damaging | Neutral | Probably damaging | Neutral |
| rs61742544 | S91T | Tolerated | Effect | C55 | Possibly Damaging | Neutral | Probably damaging | Neutral |
| rs61748836 | K857T | Tolerated | Effect | C65 | Probably Damaging | Neutral | Probably damaging | Neutral |
| rs73206291 | W208S | Tolerated | Effect | C65 | Benign | Neutral | Probably damaging | Neutral |
| rs77588184 | G628R | Deleterious | Effect | C65 | Probably Damaging | Neutral | Probably damaging | Neutral |
| rs111237660 | E1090Q | Deleterious | Neutral | C25 | Probably Damaging | Neutral | Probably damaging | Neutral |
| rs112428784 | S958F | Deleterious | Neutral | C65 | Benign | Deleterious | Probably damaging | Disease |
| **rs138534724** | **E934V** | **Deleterious** | **Effect** | **C65** | **Probably Damaging** | **Deleterious** | **Probably damaging** | **Disease** |
| **rs138713013** | **C264Y** | **Deleterious** | **Effect** | **C65** | **Probably Damaging** | **Deleterious** | **Probably damaging** | **Disease** |
| rs138770585 | L395V | Deleterious | Neutral | C25 | Benign | Neutral | Probably damaging | Neutral |
| rs141609607 | T186S | Tolerated | Effect | C55 | Benign | Neutral | Probably damaging | Neutral |
| rs141857173 | K609Q | Deleterious | Effect | C45 | Probably Damaging | Neutral | Probably damaging | Neutral |
| rs144356756 | D57G | Deleterious | Neutral | C65 | Probably Damaging | Neutral | Probably damaging | Neutral |
| rs144773361 | K331E | Deleterious | Effect | C55 | Benign | Neutral | Probably damaging | Neutral |
| rs144780663 | K644Q | Tolerated | Neutral | C45 | Benign | Neutral | Probably damaging | Neutral |
| rs144789020 | A871V | Tolerated | Neutral | C55 | Benign | Neutral | Probably damaging | Neutral |
| rs145424049 | A820D | Deleterious | Effect | C65 | Probably Damaging | Neutral | Probably damaging | Neutral |
| rs145654440 | N336D | Tolerated | Effect | C15 | Probably Damaging | Neutral | Probably damaging | Neutral |
| rs146281218 | R196K | Tolerated | Effect | C25 | Benign | Neutral | Probably damaging | Neutral |
| rs147125441 | R600G | Tolerated | Effect | C65 | Benign | Neutral | Probably damaging | Neutral |
| rs147931629 | S619N | Deleterious | Effect | C45 | Benign | Neutral | Probably damaging | Neutral |
| **rs148435996** | **S1259L** | **Deleterious** | **Effect** | **C65** | **Probably Damaging** | **Deleterious** | **Probably damaging** | **Disease** |
| rs148486769 | T193S | Tolerated | Neutral | C55 | Benign | Neutral | Probably damaging | Neutral |
| rs149380010 | E339D | Deleterious | Effect | C35 | Possibly Damaging | Neutral | Probably damaging | Neutral |
| rs150198962 | T439S | Tolerated | Neutral | C55 | Benign | Neutral | Probably damaging | Neutral |
| rs150374461 | Q1032E | Tolerated | Neutral | C25 | Possibly Damaging | Neutral | Probably damaging | Neutral |
| rs150511489 | E156D | Tolerated | Effect | C35 | Benign | Neutral | Probably damaging | Neutral |
| **rs181040560** | **E960K** | **Deleterious** | **Effect** | **C55** | **Probably Damaging** | **Deleterious** | **Probably damaging** | **Disease** |
| rs181661744 | G179R | Tolerated | Effect | C65 | Benign | Neutral | Probably damaging | Disease |
| **rs181719146** | **L1084V** | **Deleterious** | **Effect** | **C25** | **Probably Damaging** | **Deleterious** | **Probably damaging** | **Disease** |
| rs182786486 | V510I | Tolerated | Neutral | C25 | Benign | Neutral | Probably damaging | Neutral |
| rs182937751 | E680D | Deleterious | Neutral | C35 | Benign | Neutral | Probably damaging | Neutral |
| rs184656744 | S467W | Deleterious | Effect | C65 | Probably Damaging | Neutral | Probably damaging | Neutral |
| rs184851598 | M1134V | Deleterious | Neutral | C15 | Probably Damaging | Neutral | Probably damaging | Neutral |
| rs185434678 | N1005S | Tolerated | Neutral | C45 | Benign | Neutral | Probably damaging | Neutral |
| rs185663553 | T806I | Tolerated | Effect | C65 | Probably Damaging | Neutral | Probably damaging | Neutral |
| rs185762413 | K911Q | Tolerated | Effect | C45 | Probably Damaging | Neutral | Probably damaging | Neutral |
| rs186693071 | I1079T | Tolerated | Neutral | C65 | Benign | Neutral | Probably damaging | Neutral |
| rs187072799 | P503R | Deleterious | Effect | C65 | Probably Damaging | Deleterious | Probably damaging | Neutral |
| rs187103704 | N819H | Tolerated | Neutral | C65 | Benign | Neutral | Probably damaging | Neutral |
| rs187373547 | R884Q | Deleterious | Effect | C35 | Probably Damaging | Neutral | Probably damaging | Neutral |
| rs187570421 | H593L | Deleterious | Effect | C65 | Probably Damaging | Deleterious | Probably damaging | Neutral |
| rs188050011 | T412S | Tolerated | Neutral | C55 | Benign | Neutral | Probably damaging | Neutral |
| rs189274481 | E361K | Tolerated | Neutral | C55 | Benign | Neutral | Probably damaging | Neutral |
| rs189913205 | H82R | Tolerated | Neutral | C25 | Benign | Neutral | Probably damaging | Neutral |
| rs190088038 | R795Q | Tolerated | Neutral | C35 | Benign | Neutral | Probably damaging | Neutral |
| rs190516280 | P522A | Tolerated | Neutral | C25 | Benign | Neutral | Probably damaging | Neutral |
| rs190778203 | A632V | Deleterious | Neutral | C55 | Possibly Damaging | Neutral | Probably damaging | Neutral |
| rs192275444 | V405M | Tolerated | Neutral | C15 | Benign | Neutral | Probably damaging | Neutral |
| rs192325056 | Y908C | Tolerated | Effect | C65 | Benign | Deleterious | Probably damaging | Neutral |
| rs199672128 | S279L | Deleterious | Effect | C65 | Benign | Neutral | Probably damaging | Neutral |
| rs199740873 | T1012I | Deleterious | Neutral | C65 | Probably Damaging | Deleterious | Probably damaging | Disease |
| rs199774678 | A793V | Tolerated | Neutral | C55 | Benign | Neutral | Probably damaging | Neutral |
| rs199783879 | Q104H | Deleterious | Effect | C15 | Probably Damaging | Neutral | Probably damaging | Neutral |
| rs199816797 | M333I | Deleterious | Effect | C0 | Benign | Neutral | Probably damaging | Neutral |
| rs199965621 | N580K | Deleterious | Effect | C65 | Probably Damaging | Neutral | Probably damaging | Disease |
| rs200007955 | T1204M | Deleterious | Neutral | C65 | Probably Damaging | Deleterious | Probably damaging | Neutral |
| rs200082558 | Y989C | Deleterious | Effect | C65 | Possibly Damaging | Deleterious | Probably damaging | Neutral |
| rs200097535 | M163T | Deleterious | Effect | C65 | Benign | Neutral | Probably damaging | Neutral |
| rs200171947 | G718S | Tolerated | Neutral | C55 | Benign | Neutral | Probably damaging | Neutral |
| rs200247118 | M1I | Deleterious | Effect | C0 | Possibly Damaging | Neutral | Probably damaging | Neutral |
| rs200263401 | L1109F | Deleterious | Effect | C15 | Probably Damaging | Deleterious | Probably damaging | Neutral |
| rs200304323 | Q335P | Deleterious | Effect | C65 | Probably Damaging | Neutral | Probably damaging | Neutral |
| rs200439413 | L433P | Deleterious | Neutral | C65 | Benign | Neutral | Probably damaging | Neutral |
| rs200536866 | R1003Q | Deleterious | Effect | C35 | Probably Damaging | Neutral | Probably damaging | Neutral |
| rs200539556 | T591A | Deleterious | Neutral | C55 | Possibly Damaging | Neutral | Probably damaging | Neutral |
| rs200555209 | Q389R | Tolerated | Effect | C35 | Benign | Neutral | Probably damaging | Neutral |
| rs200714484 | P657L | Deleterious | Neutral | C65 | Benign | Neutral | Probably damaging | Neutral |
| rs200751032 | P302L | Deleterious | Effect | C65 | Probably Damaging | Deleterious | Probably damaging | Neutral |
| rs200756514 | G278R | Deleterious | Effect | C65 | Possibly Damaging | Neutral | Probably damaging | Neutral |
| rs200794917 | S48C | Tolerated | Neutral | C65 | Benign | Neutral | Probably damaging | Neutral |
| rs200795434 | A893V | Tolerated | Neutral | C55 | Possibly Damaging | Neutral | Probably damaging | Neutral |
| rs200869208 | Q924H | Deleterious | Neutral | C15 | Benign | Neutral | Probably damaging | Neutral |
| rs200996434 | E1148D | Tolerated | Neutral | C35 | Probably Damaging | Neutral | Probably damaging | Neutral |
| rs201091356 | Q252L | Deleterious | Effect | C65 | Possibly Damaging | Neutral | Probably damaging | Neutral |
| rs201092668 | F310L | Tolerated | Effect | C15 | Benign | Neutral | Probably damaging | Neutral |
| rs201101994 | C148Y | Tolerated | Effect | C65 | Benign | Neutral | Probably damaging | Neutral |
| **rs201397082** | **P503T** | **Deleterious** | **Effect** | **C35** | **Probably Damaging** | **Deleterious** | **Probably damaging** | **Disease** |
| rs201400993 | I1099V | Tolerated | Neutral | C25 | Benign | Neutral | Probably damaging | Neutral |
| rs201419670 | K452E | Deleterious | Effect | C55 | Benign | Neutral | Probably damaging | Neutral |
| rs201468140 | P192A | Tolerated | Effect | C25 | Possibly Damaging | Neutral | Probably damaging | Neutral |
| rs201511934 | D3N | Deleterious | Neutral | C15 | Probably Damaging | Neutral | Probably damaging | Neutral |
| rs201571326 | S610L | Tolerated | Neutral | C65 | Benign | Neutral | Probably damaging | Neutral |
| rs201616590 | I1023T | Tolerated | Neutral | C65 | Benign | Neutral | Probably damaging | Neutral |
| rs201623386 | R1019W | Deleterious | Effect | C65 | Probably Damaging | Deleterious | Probably damaging | Neutral |
| rs201642485 | T160I | Deleterious | Effect | C65 | Probably Damaging | Neutral | Probably damaging | Disease |
| **rs201647662** | **H1077L** | **Deleterious** | **Effect** | **C65** | **Probably Damaging** | **Deleterious** | **Probably damaging** | **Disease** |
| rs201829280 | A41V | Tolerated | Neutral | C55 | Benign | Neutral | Probably damaging | Neutral |
| rs201830393 | N1100S | Tolerated | Neutral | C45 | Benign | Neutral | Probably damaging | Neutral |
| rs201847623 | K932T | Deleterious | Neutral | C65 | Benign | Deleterious | Probably damaging | Neutral |
| rs201891925 | D1107N | Deleterious | Neutral | C15 | Possibly Damaging | Neutral | Probably damaging | Neutral |
| rs201951533 | V552A | Tolerated | Neutral | C55 | Benign | Neutral | Probably damaging | Neutral |
| rs201957190 | P890A | Tolerated | Neutral | C25 | Benign | Neutral | Probably damaging | Neutral |
| rs201959113 | C625W | Deleterious | Effect | C65 | Probably Damaging | Neutral | Probably damaging | Neutral |
| rs201996693 | N742S | Tolerated | Neutral | C45 | Benign | Neutral | Probably damaging | Neutral |
| rs202123481 | G194V | Tolerated | Neutral | C65 | Benign | Neutral | Probably damaging | Neutral |
| rs202148873 | F1262L | Deleterious | Effect | C15 | Probably Damaging | Deleterious | Probably damaging | Neutral |
| rs202218163 | H599Y | Tolerated | Neutral | C65 | Probably Damaging | Neutral | Probably damaging | Neutral |
| rs202225481 | K456N | Tolerated | Neutral | C65 | Benign | Neutral | Probably damaging | Neutral |
| rs367645347 | R879K | Tolerated | Neutral | C25 | Benign | Neutral | Probably damaging | Neutral |
| rs367658119 | S716F | Deleterious | Neutral | C65 | Probably Damaging | Deleterious | Probably damaging | Neutral |
| rs367826450 | C427Y | Tolerated | Effect | C65 | Benign | Deleterious | Probably damaging | Neutral |
| rs367887703 | G76D | Tolerated | Effect | C65 | Benign | Neutral | Probably damaging | Neutral |
| rs367890801 | A1126E | Tolerated | Neutral | C65 | Probably Damaging | Neutral | Probably damaging | Neutral |
| rs367941919 | P302S | Tolerated | Effect | C65 | Benign | Neutral | Probably damaging | Neutral |
| rs368080482 | I484M | Deleterious | Neutral | C0 | Benign | Neutral | Probably damaging | Neutral |
| rs368209779 | Q924L | Deleterious | Neutral | C65 | Possibly Damaging | Deleterious | Probably damaging | Neutral |
| rs368240855 | A607P | Deleterious | Effect | C25 | Possibly Damaging | Neutral | Probably damaging | Neutral |
| rs368662263 | R1006W | Deleterious | Effect | C65 | Benign | Deleterious | Probably damaging | Disease |
| rs368824729 | K468R | Deleterious | Neutral | C25 | Benign | Neutral | Probably damaging | Neutral |
| rs368852799 | P35T | Tolerated | Neutral | C35 | Benign | Neutral | Probably damaging | Neutral |
| rs368949641 | H122Y | Tolerated | Neutral | C65 | Benign | Neutral | Probably damaging | Neutral |
| rs369062985 | M653I | Tolerated | Neutral | C0 | Benign | Neutral | Probably damaging | Neutral |
| rs369235983 | S799I | Deleterious | Neutral | C65 | Probably Damaging | Neutral | Probably damaging | Neutral |
| rs369235983 | S799N | Deleterious | Neutral | C45 | Probably Damaging | Neutral | Probably damaging | Neutral |
| rs369349953 | D560G | Deleterious | Effect | C65 | Possibly Damaging | Neutral | Probably damaging | Neutral |
| rs369382976 | G334A | Deleterious | Effect | C55 | Probably Damaging | Neutral | Probably damaging | Neutral |
| rs369660389 | T556I | Deleterious | Effect | C65 | Probably Damaging | Neutral | Probably damaging | Neutral |
| rs369673100 | K1028Q | Deleterious | Neutral | C45 | Probably Damaging | Deleterious | Probably damaging | Neutral |
| rs369758225 | A821T | Tolerated | Neutral | C55 | Benign | Neutral | Probably damaging | Neutral |
| rs369762682 | T770N | Tolerated | Neutral | C55 | Benign | Neutral | Probably damaging | Neutral |
| rs370002154 | F440C | Deleterious | Effect | C65 | Probably Damaging | Neutral | Probably damaging | Neutral |
| rs370158336 | P400L | Tolerated | Neutral | C65 | Benign | Neutral | Probably damaging | Neutral |
| rs370181807 | V56I | Deleterious | Neutral | C25 | Benign | Neutral | Probably damaging | Neutral |
| rs370236573 | E626G | Deleterious | Effect | C65 | Probably Damaging | Neutral | Probably damaging | Neutral |
| **rs370363143** | **S1245Y** | **Deleterious** | **Effect** | **C65** | **Probably Damaging** | **Deleterious** | **Probably damaging** | **Disease** |
| rs370461475 | M1I | Tolerated | Effect | C0 | Possibly Damaging | Neutral | Probably damaging | Neutral |
| rs370494956 | P31L | Tolerated | Effect | C65 | Possibly Damaging | Neutral | Probably damaging | Neutral |
| rs370634502 | S318L | Tolerated | Effect | C65 | Benign | Neutral | Probably damaging | Neutral |
| rs370723597 | I420F | Tolerated | Effect | C15 | Benign | Neutral | Probably damaging | Neutral |
| rs370758570 | R1019Q | Tolerated | Neutral | C35 | Benign | Neutral | Probably damaging | Neutral |
| rs370823098 | A470G | Tolerated | Neutral | C55 | Benign | Neutral | Probably damaging | Neutral |
| rs371000006 | H315Y | Deleterious | Effect | C65 | Benign | Neutral | Probably damaging | Neutral |
| rs371016281 | A727T | Deleterious | Effect | C55 | Possibly Damaging | Neutral | Probably damaging | Neutral |
| rs371048746 | S120N | Tolerated | Neutral | C45 | Benign | Neutral | Probably damaging | Neutral |
| rs371231130 | P134S | Tolerated | Effect | C65 | Possibly Damaging | Neutral | Probably damaging | Neutral |
| rs371270782 | H391P | Tolerated | Effect | C65 | Benign | Neutral | Probably damaging | Neutral |
| rs371401804 | T584S | Tolerated | Neutral | C55 | Possibly Damaging | Neutral | Probably damaging | Neutral |
| rs371465315 | I464V | Tolerated | Neutral | C25 | Benign | Neutral | Probably damaging | Neutral |
| rs371578880 | S559F | Deleterious | Neutral | C65 | Benign | Neutral | Probably damaging | Neutral |
| rs371914626 | A820S | Tolerated | Neutral | C65 | Probably Damaging | Neutral | Probably damaging | Neutral |
| rs372141258 | T448M | Deleterious | Effect | C65 | Probably Damaging | Neutral | Probably damaging | Neutral |
| rs372276598 | W775C | Tolerated | Effect | C65 | Probably Damaging | Neutral | Probably damaging | Neutral |
| rs372284366 | M379I | Tolerated | Neutral | C0 | Benign | Neutral | Probably damaging | Neutral |
| rs372293033 | Y205C | Tolerated | Neutral | C65 | Benign | Neutral | Probably damaging | Neutral |
| rs372295537 | L1178F | Deleterious | Effect | C15 | Probably Damaging | Deleterious | Probably damaging | Neutral |
| rs372406442 | P294L | Tolerated | Neutral | C65 | Benign | Neutral | Probably damaging | Neutral |
| rs372702021 | R504S | Deleterious | Effect | C65 | Possibly Damaging | Neutral | Probably damaging | Neutral |
| rs372877313 | K1048E | Deleterious | Effect | C55 | Probably Damaging | Neutral | Probably damaging | Neutral |
| rs373001050 | P753R | Deleterious | Neutral | C65 | Probably Damaging | Deleterious | Probably damaging | Neutral |
| rs373018988 | S73R | Deleterious | Neutral | C65 | Possibly Damaging | Neutral | Probably damaging | Neutral |
| **rs373021974** | **L1143Q** | **Deleterious** | **Effect** | **C65** | **Probably Damaging** | **Deleterious** | **Probably damaging** | **Disease** |
| rs373031782 | S33L | Tolerated | Neutral | C65 | Benign | Neutral | Probably damaging | Neutral |
| rs373035733 | D162E | Deleterious | Neutral | C35 | Benign | Neutral | Probably damaging | Neutral |
| rs373040921 | K828N | Tolerated | Neutral | C65 | Benign | Neutral | Probably damaging | Neutral |
| rs373052656 | M428I | Deleterious | Neutral | C0 | Benign | Neutral | Probably damaging | Neutral |
| rs373133775 | S788C | Tolerated | Neutral | C65 | Probably Damaging | Neutral | Probably damaging | Neutral |
| rs373262842 | K644T | Deleterious | Neutral | C65 | Benign | Neutral | Probably damaging | Neutral |
| rs373295116 | P899A | Tolerated | Neutral | C25 | Benign | Neutral | Probably damaging | Neutral |
| rs373373586 | G1076V | Deleterious | Effect | C65 | Benign | Neutral | Probably damaging | Neutral |
| rs373551462 | I803T | Tolerated | Neutral | C65 | Benign | Neutral | Probably damaging | Neutral |
| rs373564089 | A218T | Tolerated | Effect | C55 | Benign | Neutral | Probably damaging | Neutral |
| rs373807625 | K881N | Deleterious | Effect | C65 | Probably Damaging | Neutral | Probably damaging | Neutral |
| rs373814039 | H119R | Deleterious | Effect | C25 | Benign | Neutral | Probably damaging | Neutral |
| rs373832107 | Q997R | Tolerated | Neutral | C35 | Probably Damaging | Neutral | Probably damaging | Neutral |
| rs374141727 | V132F | Tolerated | Neutral | C45 | Benign | Neutral | Probably damaging | Neutral |
| rs374238217 | T906A | Tolerated | Neutral | C55 | Benign | Neutral | Probably damaging | Neutral |
| rs374391254 | K437E | Tolerated | Effect | C55 | Benign | Neutral | Probably damaging | Neutral |
| rs374398513 | H954D | Tolerated | Effect | C65 | Benign | Deleterious | Probably damaging | Neutral |
| rs374407718 | P1263S | Tolerated | Neutral | C65 | Benign | Neutral | Probably damaging | Neutral |
| rs374830258 | N303S | Tolerated | Neutral | C45 | Benign | Neutral | Probably damaging | Neutral |
| rs374892928 | F172V | Deleterious | Effect | C45 | Possibly Damaging | Neutral | Probably damaging | Disease |
| rs374998389 | A470T | Tolerated | Neutral | C55 | Benign | Neutral | Probably damaging | Neutral |
| rs375028534 | R1006Q | Deleterious | Neutral | C35 | Possibly Damaging | Neutral | Probably damaging | Disease |
| rs375223669 | L855F | Tolerated | Neutral | C15 | Benign | Neutral | Probably damaging | Neutral |
| rs375223801 | P557T | Deleterious | Neutral | C35 | Possibly Damaging | Neutral | Probably damaging | Neutral |
| rs375335038 | P477R | Deleterious | Neutral | C65 | Possibly Damaging | Deleterious | Probably damaging | Neutral |
| rs375513763 | D61Y | Tolerated | Effect | C65 | Probably Damaging | Deleterious | Probably damaging | Disease |
| rs375576260 | T870S | Tolerated | Neutral | C55 | Possibly Damaging | Neutral | Probably damaging | Neutral |
| rs375637734 | T293A | Deleterious | Effect | C55 | Benign | Neutral | Probably damaging | Neutral |
| rs375638551 | D385G | Deleterious | Effect | C65 | Possibly Damaging | Neutral | Probably damaging | Neutral |
| rs375821976 | L345R | Deleterious | Effect | C65 | Possibly Damaging | Neutral | Probably damaging | Disease |
| rs375829051 | K1063N | Deleterious | Effect | C65 | Benign | Deleterious | Probably damaging | Neutral |
| rs375889062 | P898S | Tolerated | Neutral | C65 | Benign | Neutral | Probably damaging | Neutral |
| rs376191670 | I260V | Tolerated | Neutral | C25 | Benign | Neutral | Probably damaging | Neutral |
| rs376446886 | A447V | Tolerated | Neutral | C55 | Benign | Neutral | Probably damaging | Neutral |
| rs376698024 | T26R | Deleterious | Effect | C65 | Probably Damaging | Neutral | Probably damaging | Neutral |
| rs376759161 | I659V | Tolerated | Neutral | C25 | Benign | Neutral | Probably damaging | Neutral |
| rs376884759 | S227R | Tolerated | Neutral | C65 | Benign | Neutral | Probably damaging | Neutral |
| rs376983412 | A1026G | Deleterious | Neutral | C55 | Probably Damaging | Deleterious | Probably damaging | Neutral |
| rs377130971 | Y1015H | Tolerated | Neutral | C65 | Probably Damaging | Neutral | Probably damaging | Neutral |
| rs377188798 | S1249F | Tolerated | Effect | C65 | Probably Damaging | Deleterious | Probably damaging | Neutral |
| rs377191713 | S42C | Deleterious | Effect | C65 | Probably Damaging | Neutral | Probably damaging | Neutral |
| rs377197772 | T436A | Tolerated | Neutral | C55 | Benign | Neutral | Probably damaging | Neutral |
| rs377257691 | D376N | Deleterious | Neutral | C15 | Possibly Damaging | Neutral | Probably damaging | Neutral |
| rs377310381 | A415S | Tolerated | Neutral | C65 | Possibly Damaging | Neutral | Probably damaging | Neutral |
| rs377451974 | V630I | Deleterious | Neutral | C25 | Probably Damaging | Neutral | Probably damaging | Neutral |
| rs377477468 | E626K | Deleterious | Effect | C55 | Probably Damaging | Neutral | Probably damaging | Neutral |
| rs377509441 | E826G | Deleterious | Effect | C65 | Possibly Damaging | Neutral | Probably damaging | Neutral |
| rs377602735 | T216M | Tolerated | Neutral | C65 | Benign | Neutral | Probably damaging | Neutral |

**S1 Table. Identification of the impact of nsSNPs of the *MTUS1* gene**
