# Supplementary material for: In silico analysis of deleterious SNPs of human MTUS1 gene and their impacts on subsequent protein structure and function
Source: PLoS One. 2021 Jun 14;16(6):e0252932. doi: 10.1371/journal.pone.0252932 (PMC8202925; doi:10.1371/journal.pone.0252932)
Supplement: S2 Table — (DOCX) [file pone.0252932.s007.docx]

**S2 Table: Validation scores of different computational tools for the models from I-TASSER**

| **I-TASSER**  **Model** | **C-score** | **SWISS-MODEL Ramachandran Favorable region (%)** | **PROCHECK**  **Core region of Ramachandran**  **Plot (%)** | **QMEAN**  **Score** | **Molprobity**  **Score** | **ERRAT**  **Score** |
| --- | --- | --- | --- | --- | --- | --- |
| 01 | -1.67 | 69.18 | 64.4 | -11.74 | 3.49 | 84.6645 |
| 02 | -3.56 | 63.79 | 64.4 | -11.74 | 3.24 | 84.7078 |
| 03 | -3.86 | 54.98 | 55.4 | -15.12 | 3.47 | 73.0463 |
| 04 | -4.11 | 46.39 | 46.3 | -18.09 | 3.59 | Error |
| 05 | -4.23 | Failed | 49.3 | -16.60 | Failed | Error |
